# Hippo signaling effectors YAP and TAZ induce Epstein-Barr Virus (EBV) lytic reactivation through TEADs in epithelial cells

**DOI:** 10.1371/journal.ppat.1009783

**Published:** 2021-08-02

**Authors:** Nicholas Van Sciver, Makoto Ohashi, Nicholas P. Pauly, Jillian A. Bristol, Scott E. Nelson, Eric C. Johannsen, Shannon C. Kenney

**Affiliations:** 1 Department of Oncology, School of Medicine and Public Health, University of Wisconsin- Madison, Madison, Wisconsin, United States of America; 2 Cellular and Molecular Pathology Graduate Training Program, University of Wisconsin- Madison, Madison, Wisconsin, United States of America; 3 Department of Medicine, School of Medicine and Public Health, University of Wisconsin-Madison, Madison, Wisconsin, United States of America; University of North Carolina at Chapel Hill, UNITED STATES

## Abstract

The Epstein-Barr virus (EBV) human herpesvirus is associated with B-cell and epithelial-cell malignancies, and both the latent and lytic forms of viral infection contribute to the development of EBV-associated tumors. Here we show that the Hippo signaling effectors, YAP and TAZ, promote lytic EBV reactivation in epithelial cells. The transcriptional co-activators YAP/TAZ (which are inhibited by Hippo signaling) interact with DNA-binding proteins, particularly TEADs, to induce transcription. We demonstrate that depletion of either YAP or TAZ inhibits the ability of phorbol ester (TPA) treatment, cellular differentiation or the EBV BRLF1 immediate-early (IE) protein to induce lytic EBV reactivation in oral keratinocytes, and show that over-expression of constitutively active forms of YAP and TAZ reactivate lytic EBV infection in conjunction with TEAD family members. Mechanistically, we find that YAP and TAZ interact with, and activate, the EBV BZLF1 immediate-early promoter. Furthermore, we demonstrate that YAP, TAZ, and TEAD family members are expressed at much higher levels in epithelial cell lines in comparison to B-cell lines, and find that EBV infection of oral keratinocytes increases the level of activated (dephosphorylated) YAP and TAZ. Finally, we have discovered that lysophosphatidic acid (LPA), a known YAP/TAZ activator that plays an important role in inflammation, induces EBV lytic reactivation in epithelial cells through a YAP/TAZ dependent mechanism. Together these results establish that YAP/TAZ are powerful inducers of the lytic form of EBV infection and suggest that the ability of EBV to enter latency in B cells at least partially reflects the extremely low levels of YAP/TAZ and TEADs in this cell type.

## Author summary

EBV causes infectious mononucleosis and persists in latently infected B cells for life. The virus periodically reactivates to a lytic form that produces infectious virus particles, allowing the virus to infect new cells and be transmitted to new hosts. Oral epithelial cells are an important site of lytic viral infection, promoting secretion of infectious virus into saliva. Rarely, EBV infection results in B-cell and epithelial-cell human tumors, and both the latent and lytic forms of infection contribute to these malignancies. However, the viral and cellular factors that determine whether the virus remains latent or lytic are still incompletely understood. Here we have discovered that the Hippo signaling pathway is an important regulator of lytic EBV reactivation, particularly in epithelial cells. We show that transcriptional co-activators YAP and TAZ (which are turned off by Hippo signaling) are strong inducers of lytic EBV reactivation, collaborating with DNA-binding TEAD proteins to activate EBV immediate-early genes. Furthermore, we demonstrate that epithelial cells express much higher levels of YAP/TAZ/TEADs in comparison to B cells, helping to explain why B cells support latent infection. Finally, we find that lysophosphatidic acid, a YAP/TAZ activator, induces lytic reactivation through a YAP/TAZ-dependent mechanism.

## Introduction

Epstein-Barr virus (EBV) is a gamma herpesvirus that causes the clinical syndrome, infectious mononucleosis, and infects over 90% of the human population. EBV primarily infects B cells and oropharyngeal epithelial cells. While the vast majority of EBV-infected individuals experience no additional symptoms after recovery from the initial viral infection, a minority of individuals go on to develop EBV-associated B-cell and epithelial-cell cancers such as Burkitt lymphoma (BL), Hodgkin lymphoma, nasopharyngeal carcinoma (NPC), and gastric carcinoma [1–4].

Like all herpesviruses, EBV can infect host cells in either latent or lytic forms. During latency, EBV expresses relatively few genes, and the latency proteins produced enable the viral genome to persist and the infected cell to survive. However, once lytic replication is initiated EBV’s full gene complement is expressed, and infectious virions are produced [5,6]. EBV persists in the memory B-cell population in a tightly latent form for the life of the host, but can periodically reactivate to the lytic form of viral infection when B cells are stimulated by antigen and/or differentiate into plasma cells. In contrast, EBV-infected epithelial cells in the oropharynx generally undergo lytic replication and shed virus into the saliva [7–9].

Worldwide, the number of EBV-infected epithelial cell tumors greatly exceeds the number of EBV-infected B cell tumors [3]. The underlying molecular mechanisms that control EBV lytic reactivation, particularly in epithelial cells, remain incompletely characterized. Although excessive lytic EBV infection may play an important early role in promoting EBV-induced tumors by increasing the total number of EBV-infected cells, fully formed tumors are largely maintained by latent EBV infection [10–14]. Indeed, suppression of excessive lytic EBV infection (which generally kills the host cell) is likely required for the development of these tumors. Therefore, a better understanding in regard to mechanisms by which cellular and viral factors regulate the latent-to-lytic EBV switch at different time points during tumor development may provide insights for preventing or controlling these malignancies. For example, inducing EBV lytic replication in latently infected tumor cells has been proposed as a method for specifically blocking the growth of EBV-infected malignancies [15–17].

EBV switches from the latent to the lytic form of infection when cellular transcription factors activate the EBV immediate-early (IE) promoters, BZLF1 (Zp) and BRLF1 (Rp) [6,18–20]. The BZLF1 (Z) and BRLF1 (R) IE proteins are viral transcription factors that collaboratively induce expression of the EBV early lytic genes that encode proteins required for lytic viral replication [6,21–23]. Following viral DNA replication, expression of the EBV late lytic genes, which encode structural viral proteins, occurs, allowing release of infectious virion particles. Z preferentially binds and activates promoters with methylated DNA, whereas R preferentially activates promoters with unmethylated (or 5-hydroxymethylated) DNA [24–26]. Z expression efficiently initiates lytic EBV reactivation in B cell lines (in which the viral genome becomes highly methylated), while R expression is required for initiation of lytic reactivation in the EBV-infected telomerase-immortalized oral keratinocyte (NOKs) cell line, in which the viral genome remains hypomethylated [6,24,27,28]. Regardless of cell type, once expressed the Z and R proteins activate each other’s promoters, and both proteins are required for expression of most lytic viral genes and viral genome replication.

A number of different cellular stimuli (including cellular differentiation, hypoxia, B cell receptor engagement, DNA damage and caspase activation) can initiate lytic EBV infection [18–20,29–34], while other factors (including CAF1, HIRA, myc, ZEB 1 and ZEB2) can inhibit it [35–38]. Two master regulators of stratified epithelial cell differentiation, KLF4 and BLIMP1, synergistically induce lytic EBV reactivation in differentiated cells by collaboratively activating the Zp and Rp IE promoters [18,19]. Nevertheless, lytic EBV infection also occurs in a small subset of undifferentiated NPC tumor cells and may even contribute to the development of this cancer type [10]. Two recent studies examining EBV infection of pseudostratified primary nasopharyngeal respiratory epithelial cells grown in air-liquid interface culture (which may be a good model for early NPC lesions) found predominantly latent viral infection in the undifferentiated basal epithelial cell layer, and predominantly lytic infection in suprabasal layers [39,40]. However, there was no obvious association between high level KLF4 and BLIMP1 expression and lytic EBV gene expression in this model [40], and some lytic gene expression was found in the undifferentiated basal cells [39]. Thus, the cellular transcription factors important for inducing lytic EBV infection in undifferentiated and differentiated epithelial cells have not yet been fully defined.

Yes-associated protein (YAP) and its paralog TAZ (also known as WWTR1) are the Hippo signaling pathway’s transcriptional effector genes. YAP and TAZ are considered oncogenes and are overexpressed or amplified in numerous epithelial cancers [41–45]. Nevertheless, the effects of YAP and TAZ are highly tissue-specific and context-dependent, as in some cases YAP and TAZ have been reported to enhance cellular differentiation [46–48]. The tumor suppressor Hippo signaling pathway negatively regulates YAP/TAZ function via activation of the LATS1/2 kinases, which phosphorylate YAP/TAZ at multiple sites [41,49–52], allowing YAP/TAZ to interact with 14-3-3 proteins (sequestering YAP/TAZ in the cytoplasm) and be degraded by the proteasome [41,49,53]. YAP/TAZ dephosphorylation, which can be induced by many different types of stimuli, results in localization of these proteins to the nucleus, where YAP/TAZ can activate gene expression. YAP and TAZ do not contain a DNA-binding domain and require a DNA-bound cofactor (most commonly one of the four TEAD family members) to activate gene expression [46,54–56]. Transcription factors such as p73, ErbB4, KLF4, SMAD 2/3 and MRTF can also facilitate YAP/TAZ transcriptional activities [46,53–55,57–61].

Here we show that both YAP and TAZ are potent activators of the EBV lytic cascade. We find that loss of constitutive YAP or TAZ expression reduces differentiation-induced or phorbol ester-induced lytic EBV reactivation in epithelial cell lines and inhibits BRLF1-mediated reactivation in EBV-infected NOKs cells. Conversely, we show that over-expression of either constitutively active YAP or TAZ induces lytic reactivation in EBV-infected epithelial cell lines and demonstrate that YAP and TAZ cooperate with TEAD family members to activate the Z IE promoter. Furthermore, we find that YAP, TAZ and TEAD proteins are expressed at high levels in epithelial cell lines, but not B-cell lines, and show that EBV infection enhances YAP/TAZ activity in NOKs cells. Finally, we have discovered that lysophosphatidic acid (LPA), a known YAP/TAZ activator that plays an important role in inflammation, induces EBV lytic reactivation in epithelial cells through a YAP/TAZ dependent mechanism. These results reveal that YAP and TAZ are novel inducers of lytic EBV reactivation in epithelial cells.

## Results

### YAP expression is essential for constitutive lytic protein expression in an EBV-infected AGS gastric carcinoma cell line

**Fig 1:**
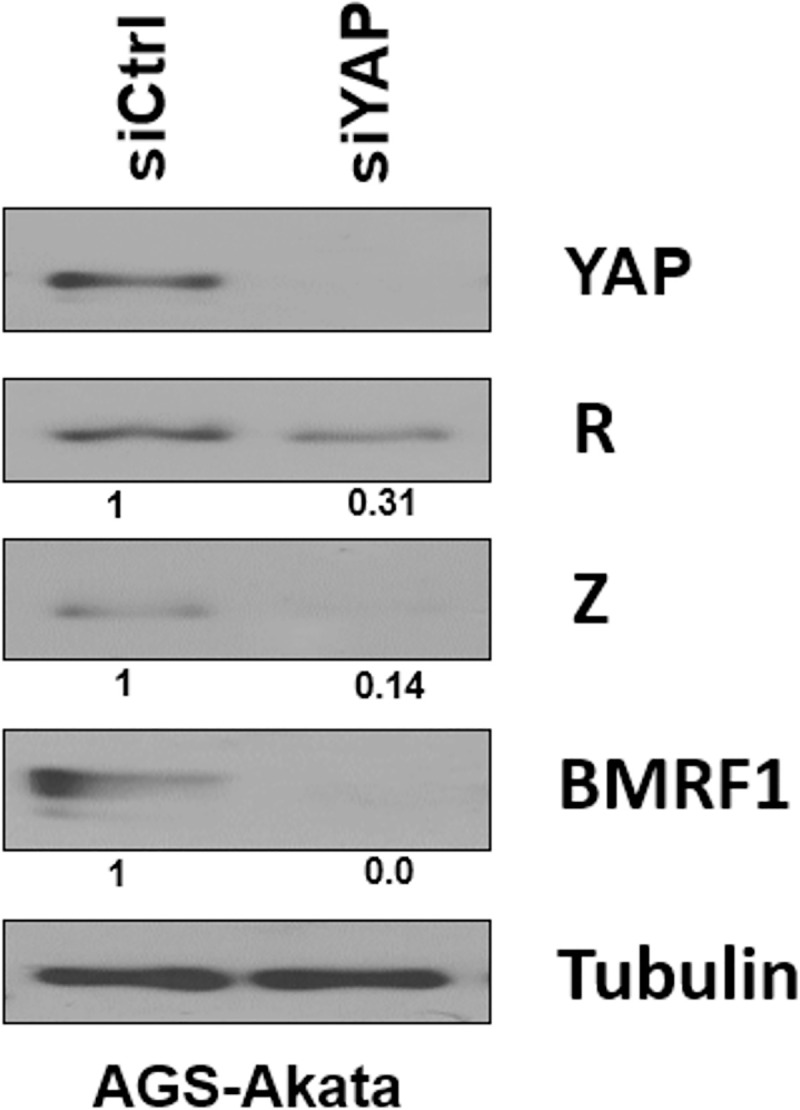
Depletion of YAP decreases constitutive lytic protein expression in AGS-Akata cells. Pooled siRNAs targeting YAP or a control sequence were transfected into AGS-Akata cells. Cells were harvested after two days and immunoblotted for Z, R, BMRF1, YAP and tubulin (loading control). Results were quantitated using ImageJ software; results were normalized to the tubulin result for each condition, with the siRNA control result set as 1.

Since we previously showed that AGS gastric carcinoma cells stably infected with the Akata EBV strain (AGS-Akata cells) have an unusually high level of lytic infection [62], and gastric carcinomas (including AGS) often express constitutively active YAP [63,64], we asked whether YAP expression is required for constitutive lytic EBV protein expression in these cells. AGS-Akata cells were treated with control siRNA or siRNA targeting YAP and the level of various lytic EBV proteins was examined two days later using immunoblot analysis. As shown in Fig 1, knockdown of YAP expression resulted in decreased expression of the Z and R immediate-early lytic EBV proteins as well as the BMRF1 early lytic protein. A similar result was obtained in another experiment (S1 Fig). These results suggest that activated YAP is required for efficient lytic EBV protein expression in AGS-Akata cells.

### YAP and TAZ are both required for the ability of the phorbol ester, TPA, to induce EBV lytic reactivation in NOKs-Akata cells

**Fig 2:**
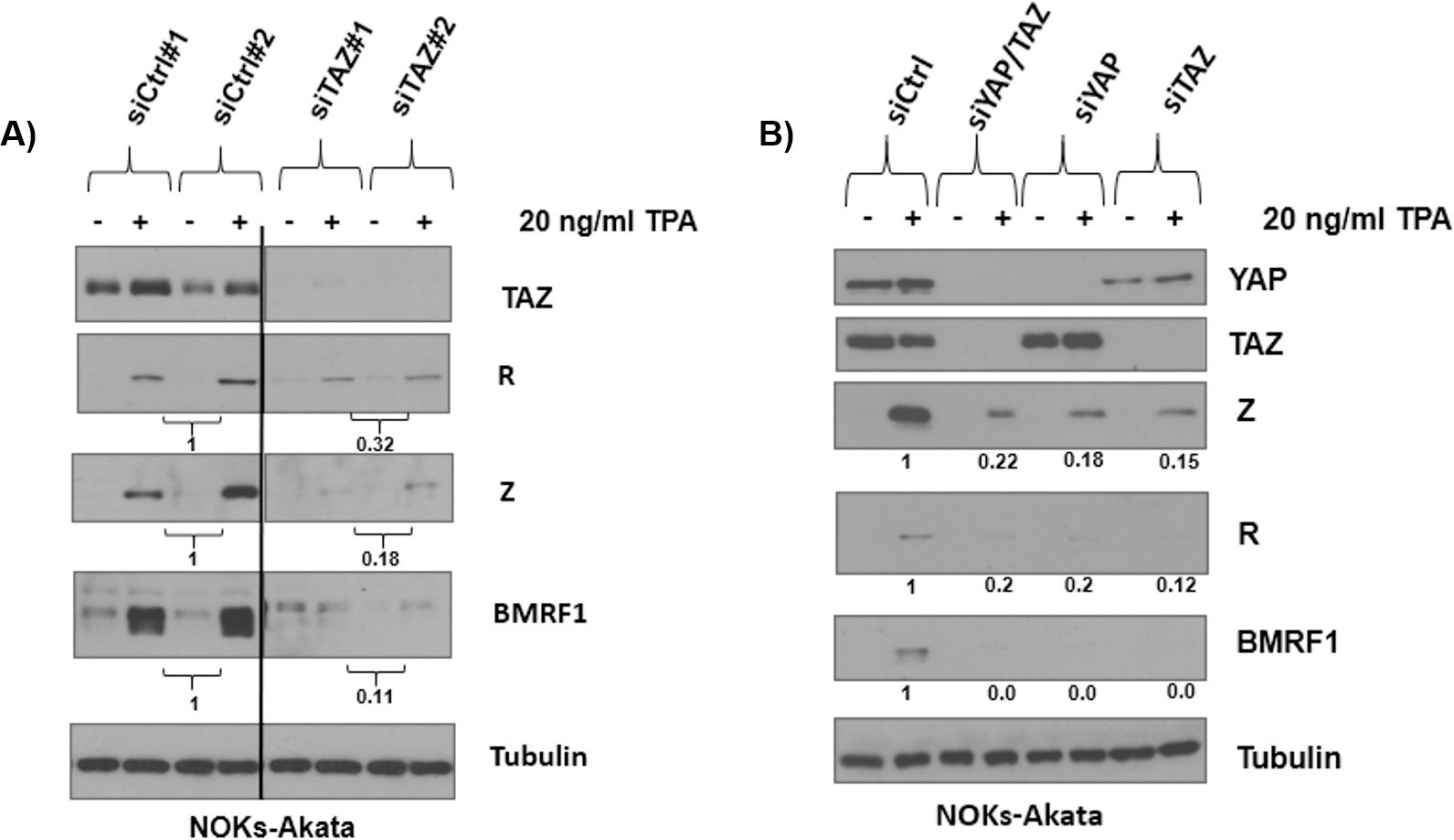
YAP and TAZ expression are both essential for efficient TPA-induced EBV lytic reactivation in NOKs cells. NOKs-Akata cells were treated with either **A)** 20 pM of two different control siRNAs, or 20 pM of two different siRNAs against TAZ or **B)** 20 pM of control siRNA, or 10 pM of YAP and 10 pM TAZ pooled siRNAs (combined), or 20 pM pooled siRNAs against either YAP or TAZ. 24 hours post-siRNA treatment, the cells were dosed with 20 ng/ml TPA. After a subsequent 24 hours the cells were harvested for immunoblots, where the expression of YAP, TAZ, Z, R and BMRF1 was determined. Tubulin was used as a loading control. In figure A, results were quantitated using ImageJ software, with the results for each set of siRNAs averaged and then normalized to the tubulin result for each condition. The average control siRNA result was set as 1. In figure B, quantitated results were normalized to the tubulin result for each condition, and the siRNA control result was set as 1. Black line indicates where irrelevant lanes in the western blot were removed.

The phorbol ester TPA is a well-characterized inducer of EBV lytic reactivation, and has been previously shown to induce YAP dephosphorylation and activation in a cell type-dependent manner by dephosphorylating LATS and inhibiting their function [65–67]. To determine if YAP and/or TAZ is required for TPA to induce lytic reactivation of EBV-infected NOKs cells, NOKs cells stably infected with Akata strain EBV (NOKs-Akata cells) were treated for 24 hours with control siRNAs or siRNAs targeting YAP and/or TAZ, and then treated with or without TPA for another 24 hours before harvesting cells for immunoblot analysis. As shown in Fig 2A and 2B, knockdown of either YAP or TAZ decreased the ability of TPA to induce expression of the EBV Z, R, and BMRF1 proteins. We confirmed that YAP and TAZ were depleted in their respective conditions as expected, although in some cases knockdown of TAZ also reduced YAP expression to a lesser extent (suggesting that TAZ may enhance YAP expression in these cells). Interestingly, depletion of either YAP or TAZ alone was as effective at inhibiting TPA-induced lytic reactivation in NOKs cells as YAP/TAZ double knockdown (Fig 2B). Similar results were obtained in another experiment (S2 Fig). These results demonstrate that the ability of TPA to induce lytic EBV reactivation in NOKs cells requires both YAP and TAZ, suggesting that YAP and TAZ each contribute independently to TPA-induced lytic reactivation.

### YAP and TAZ are both required for differentiation-induced EBV lytic reactivation in NOKs cells

**Fig 3:**
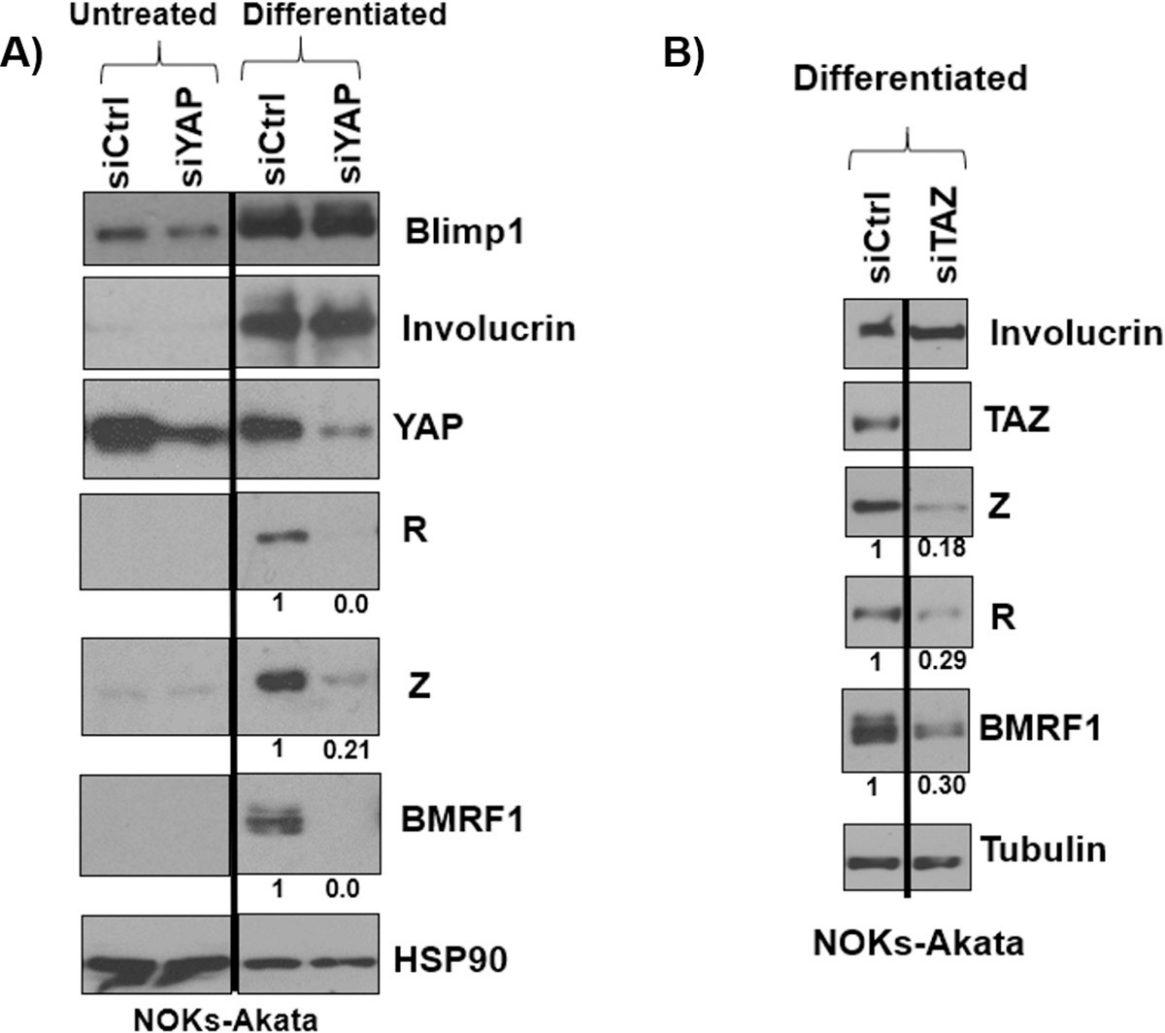
Depletion of YAP or TAZ inhibits EBV lytic reactivation during epithelial cell differentiation. NOKs-Akata cells were transfected with pooled siRNAs targeting YAP or a control siRNA **A)** or **B)** transfected with pooled siRNAs targeting TAZ or a control siRNA. One day after transfection the cells were plated onto type I and type III collagen treated membranes, where they were transfected again with the same siRNAs. The cells were then lifted to the air-liquid interface where they remained for two days before harvesting for an immunoblot to determine the expression levels of BLIMP1, involucrin, YAP, TAZ, Z, R, BMRF1, HSP90 or tubulin as a loading control as indicated. Results were quantitated using ImageJ software and normalized to the loading control result for each condition. The siRNA control result was set as 1. Black lines indicate where irrelevant lanes in the western blot were removed.

We next asked if both YAP and TAZ are required for the ability of EBV to lytically reactivate during epithelial cell differentiation. NOKs-Akata cells were treated for 24 hours with either control siRNA or siRNAs directed against YAP or TAZ, plated on collagen treated membranes for another 24 hours, and then transfected again with the various siRNAs and lifted to the air-liquid interface to induce differentiation before harvesting 48 hours later for immunoblot analysis. As shown in Fig 3, NOKs-Akata cells treated with control siRNA differentiated (as determined by expression of the differentiation proteins involucrin and BLIMP1) and lytically reactivated (as determined by expression of the lytic EBV proteins, Z, R and BMRF1). NOKs-Akata cells treated with YAP siRNA or TAZ siRNA had decreased expression of the lytic EBV proteins Z, R, and BMRF1 (Fig 3A and 3B), although expression of the differentiation markers involucrin and BLIMP1 was not altered. These results suggest that YAP and TAZ also facilitate EBV lytic reactivation during epithelial cell differentiation although they are not required for differentiation of this cell type per se.

### BRLF1-mediated disruption of latency in NOKs-Akata cells also requires YAP and TAZ expression

**Fig 4:**
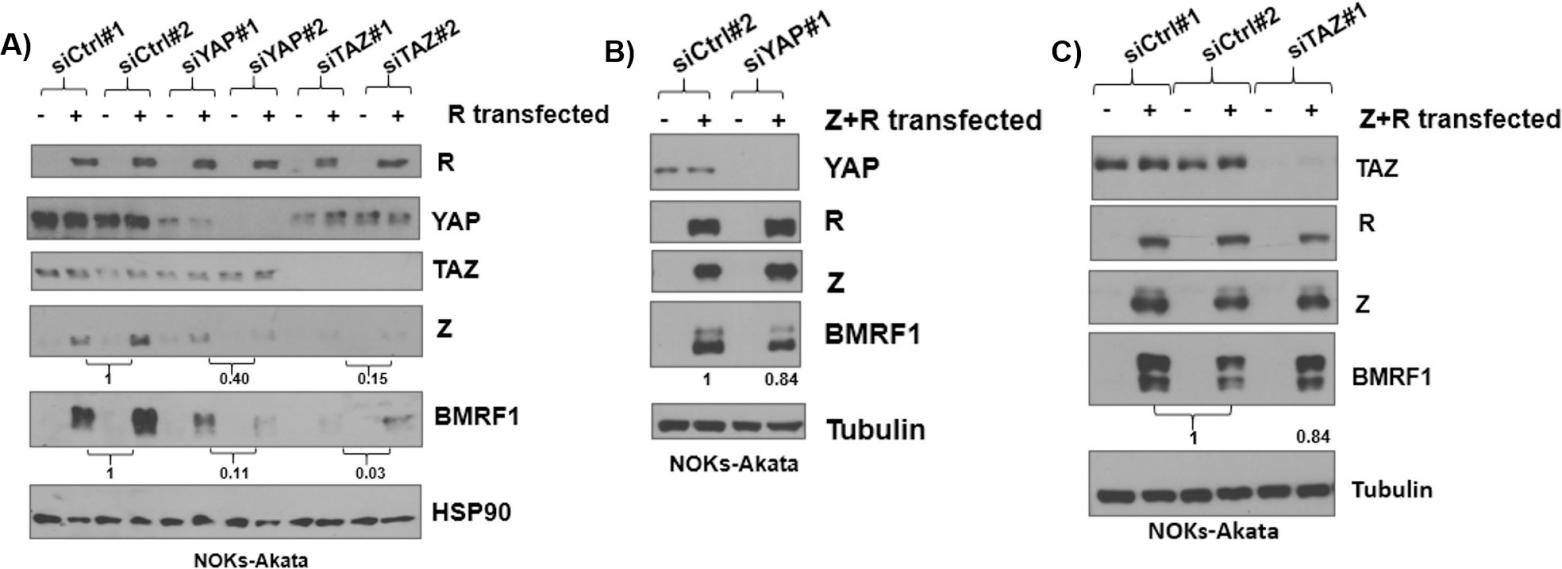
Efficient R-induced lytic reactivation requires expression of YAP and TAZ. **A)** NOKS-Akata cells were transfected with control siRNA or siRNA against YAP or TAZ, and then 24 hours later cells were transfected with an R expression vector or control vector. After another 24 hours an immunoblot was performed to assess the levels of R, YAP, TAZ, Z, BMRF1, and HSP90. **B**) NOKS-Akata cells were transfected with control siRNA or siRNA against YAP, and 24 hours later were transfected with both Z and R expression vectors. A western blot was performed to determine expression levels of YAP, Z, R, BMRF1 and tubulin. **C**) NOKS-Akata cells were transfected with control siRNA or siRNAs against TAZ, and were then transfected 24 hours later with Z and R expression vectors. The cells were harvested after 24 hours for western blot analysis to detect expression of TAZ, R, Z, BMRF1, and tubulin. Results were quantitated using ImageJ software and normalized to the loading control result for each condition. The siRNA control result was set as 1.

We previously showed that over-expression of the R IE protein, but not the Z IE protein, can initiate EBV lytic reactivation in NOKs-Akata cells [24,25]. During R-mediated viral reactivation, R initially activates expression of the Z IE gene, and then the R and Z proteins together collaboratively induce expression of the early lytic EBV genes. To assess if YAP and/or TAZ facilitate R induction of lytic reactivation, we treated NOKs-Akata cells with either YAP, TAZ or control siRNAs, and transfected cells 24 hours later with an R expression vector or empty vector control, and then performed immunoblot analysis at 24 hours after R transfection. We found that the depletion of either YAP or TAZ did not affect the level of transfected R protein, but greatly reduced the ability of R to induce the expression of the Z or BMRF1 lytic proteins (Fig 4A). Similar results were obtained in other experiments (S3 Fig). Since induction of BMRF1 early lytic gene expression requires both the Z and R proteins, these results suggest that YAP and TAZ are required for efficient R-mediated activation of the Z IE promoter. Consistent with this, we found that YAP and TAZ knockdown did not significantly inhibit the ability of transfected Z and R proteins together to induce BMRF1 expression (Figs 4B, 4C and S3).

### Constitutively activated YAP and TAZ are sufficient to induce EBV lytic reactivation in epithelial cell lines

**Fig 5:**
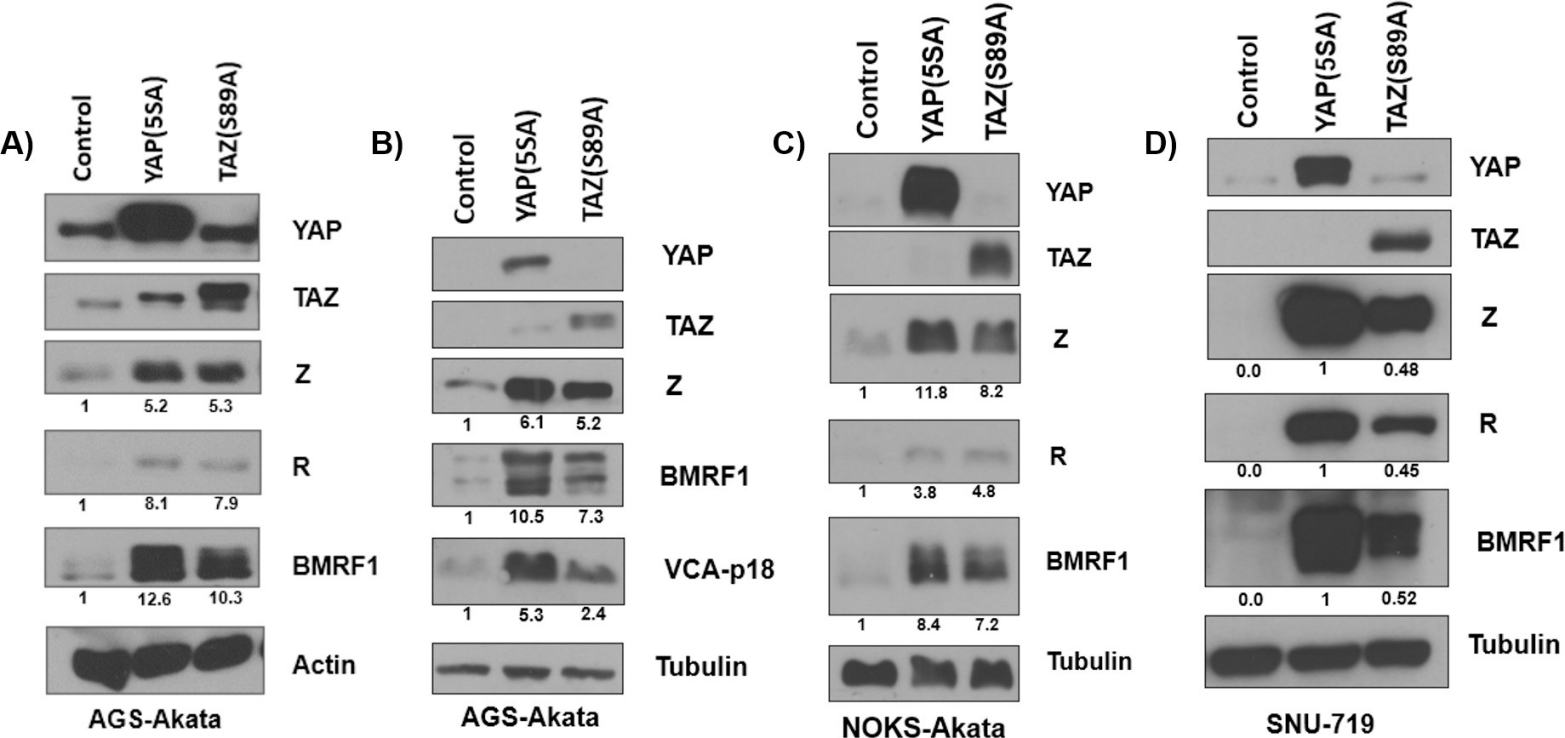
Constitutively activated YAP and TAZ induce lytic EBV reactivation in epithelial cell lines. **A)** AGS-Akata cells were transfected with constitutively active YAP (YAP(5SA)), or TAZ (TAZ(S89A)) expression vectors, or a vector control. Two days after transfection, the cells were harvested for a western blot and the expression levels of YAP, TAZ, Z, R, BMRF1, and actin (loading control) was examined. **B)** AGS-Akata cells were transfected with constitutively active YAP(5SA), TAZ(S89A), or a vector control. Three days after transfection the cells were harvested for an immunoblot where the expression of YAP, TAZ, Z, BMRF1, VCA-p18 and tubulin was determined. **C)** NOKs-Akata cells were transfected with YAP(5SA) or TAZ(S89A) expression vectors, or a vector control, and immunoblot was performed to detect expression of transfected proteins YAP and TAZ, and Z, R, BMRF1, and tubulin (loading control). **D)** SNU-719 gastric carcinoma cells were transfected with either YAP(5SA), TAZ(S89A), or control expression vectors. Two days after transfection the cells were harvested for a western blot, and the expression of YAP, TAZ, Z, R, BMRF1, and tubulin was assessed. Results were quantitated using ImageJ software and normalized to the loading control result for each condition. The vector control or YAP(5SA) result (if vector control had no signal) was set as 1.

We next asked if over-expression of constitutively active forms of YAP and/or TAZ is sufficient to induce lytic EBV reactivation in epithelial cells. AGS-Akata cells were transfected with expression vectors for constitutively active forms of YAP (YAP(5SA)) or TAZ (TAZ (S89A)) that are each missing specific serine residues that inhibit YAP and TAZ nuclear translocation when phosphorylated by LATS1/2 [51,52,54,55]. As shown in Fig 5A and 5B, both YAP(5SA) and TAZ(S89A) strongly induced expression of the EBV immediate-early and early lytic proteins Z, R, and BMRF1, and the late lytic viral protein VCA-p18, compared to the vector control. Similar results were found in NOKs-Akata cells (Fig 5C). Both YAP(5SA) and TAZ(S89A) also induced expression of the EBV lytic proteins Z, R, and BMRF1 in the EBV-infected SNU-719 gastric carcinoma cell line (which was EBV positive in the original patient tumor and has remained EBV-positive in culture [68](Figs 5D and S4C). These findings indicate that YAP and TAZ (when they are not phosphorylated by LATS and thus are transcriptionally active) are indeed sufficient to induce EBV lytic reactivation in both gastric and oral epithelial cell types.

### YAP cooperates with TEAD family members to induce lytic EBV reactivation in epithelial cells

**Fig 6:**
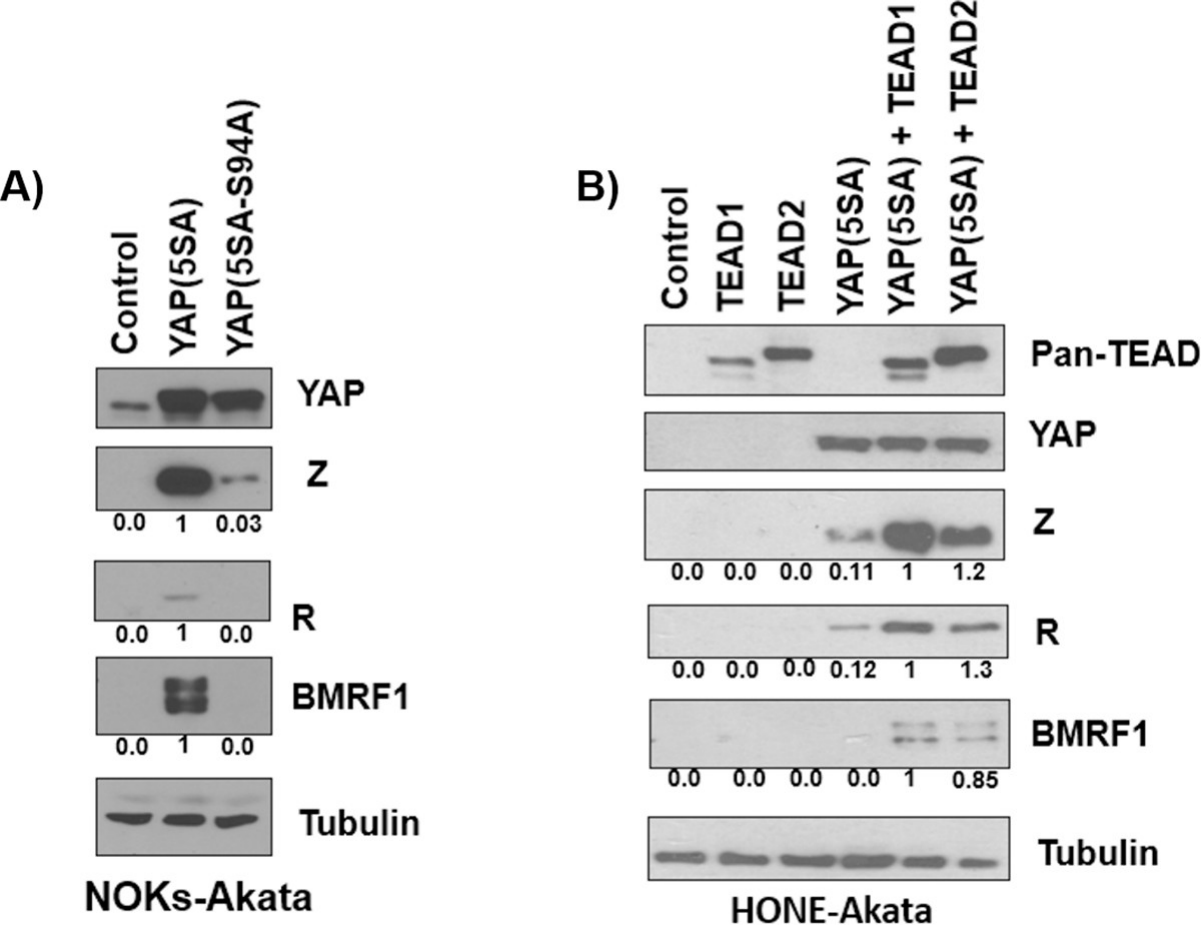
YAP and TAZ cooperate with TEADs to induce EBV lytic reactivation in epithelial cells. **A)** NOKs-Akata cells were transfected with YAP(5SA-S94A), YAP(5SA), or control vectors. After 48 hours an immunoblot was performed to detect expression of Z, R, BMRF1, YAP, and tubulin. Results were quantitated using ImageJ software and normalized to the loading control result for each condition; the YAP(5SA) vector result was set as 1. **B)** HONE-Akata cells were transfected with the YAP(5SA) expression vector with or without a TEAD1 or TEAD2 expression vector. 48 hours after transfection an immunoblot was performed to examine the expression of Z, R, BMRF1, TEADs and a tubulin loading control. Results were quantitated using ImageJ software and normalized to the loading control result for each condition. The YAP(5SA) vector plus TEAD1 result was set as 1.

YAP and TAZ commonly cooperate with TEAD family members to activate target genes, as both YAP and TAZ lack a DNA binding domain and cannot access the DNA without a DNA-binding partner [54,55,59,69]. To determine if YAP cooperates with TEADs to induce EBV lytic reactivation, we transfected NOKs-Akata cells with either the constitutively active YAP(5SA) vector or a mutant form of this vector (YAP(5SA-S94A)) that specifically prevents YAP from interacting with TEAD family members [54]. We found that the YAP(5SA-S94A) protein is deficient in inducing lytic EBV reactivation in comparison to the YAP(5SA) vector (Figs 6A and S5A).

To determine if over-expression of TEADs synergizes with YAP to induce lytic EBV reactivation, we transfected HONE-Akata cells with the constitutively active YAP vector in the presence or absence of co-transfected expression vectors for TEAD1 or TEAD2. We found that while YAP induces EBV lytic gene expression by itself, the lytic inducing effect is much stronger when YAP is co-transfected with either TEAD1 or TEAD2 expression vectors (Figs 6B and S5B). These results suggest that YAP and TEADs collaborate to activate lytic EBV protein expression.

### YAP and TAZ induce Z IE promoter activity

**Fig 7:**
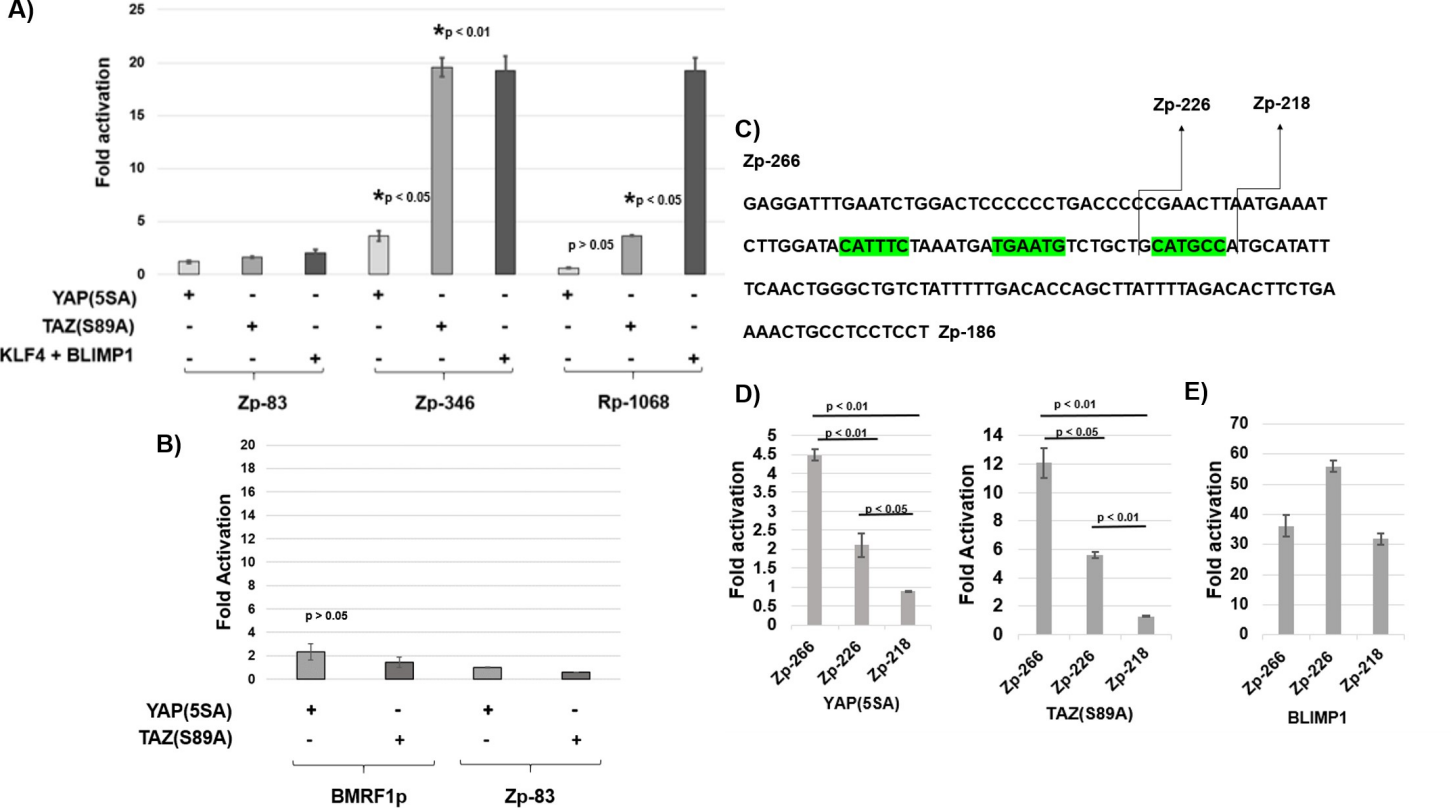
YAP and TAZ induce Z promoter activity through TEAD motifs located from -218 to -251. **A)** EBV negative HeLa cells were transfected with luciferase promoter constructs that were driven by the intact Z promoter or R promoter (containing 346 or 1068 bp, respectively, of each promoter sequence relative to the transcriptional start site), or a negative control promoter (containing only 83 bp of Zp promoter sequence), along with either a vector control, or YAP(5SA), TAZ(S89A), or KLF4+BLIMP1 expression vectors. The luciferase activity produced by each promoter was measured 48 hours post-transfection. The average fold difference in luciferase activity in conditions transfected with control vector versus YAP, TAZ or KLF4/BLIMP1 vectors is shown, along with the standard error. Statistical analysis (two-sample t-test) showed results of luciferase activity of the Zp-346 vector transfected with YAP(5SA) or TAZ(S89A) vectors, versus the vector control, were significantly different as indicated. **B)** HeLa cells were transfected with luciferase promoter constructs that were driven by the early lytic BMRF1 promoter or the negative control promoter (Zp-83) along with either a vector control, or YAP(5SA) or TAZ(S89A) vectors. The average fold difference in luciferase activity in conditions transfected with control vector versus YAP or TAZ vectors is shown, along with the standard error and p value. **C)** The sequence of the BZLF1 (Zp) promoter located between -266 and -186 (relative to the transcriptional start site) is shown; suspected TEAD binding motifs, each containing a 5/6 match to the consensus TEAD binding site (CATTCC) are outlined in green. Arrows indicate locations of the 5’ Z promoter deletions. **D)** EBV negative HeLa cells were transfected with 5’ luciferase Z promoter constructs with either a vector control or, the YAP(5SA) expression vector, the TAZ(S89A) expression vector, or a BLIMP1 expression vector as indicated. The average fold difference in luciferase activity in conditions transfected with control vector versus the YAP(5SA), TAZ(S89A), or BLIMP1 expression vectors is shown, along with the standard error. Statistical analysis for YAP(5SA) and TAZ(S89A) induction of the Z promoter constructs -266, -226, -218, was done with the two-sample t-test. **E)** EBV negative HeLa cells were transfected with 5’ luciferase Z promoter constructs with either a vector control or a BLIMP1 expression vector as indicated.

To determine if YAP and/or TAZ can activate the Z (Zp) or R (Rp) IE promoters in reporter gene assays, we transfected HeLa cells with Zp (Zp-346) or Rp (Rp-1068) promoter constructs (driving luciferase gene expression) in the presence of absence of co-transfected constitutively active YAP or TAZ expression vectors and performed luciferase assays. Co-transfection with the TAZ(S89A) vector induced the activity of the Zp-346 promoter, and to a lesser extent the Rp-1068 promoter, while not affecting the negative control Zp-83 promoter vector. Co-transfection with the YAP(5SA) vector also activated the Zp-346 promoter construct (although the effect was weaker than that of the TAZ vector) but did not activate the Rp or Zp-83 promoters (Fig 7A). In contrast, YAP and TAZ did not significantly activate the early lytic BMRF1 EBV promoter in EBV-negative cells (Fig 7B). As previously reported by our group, the combination of co-transfected KLF4 and BLIMP1 also potently induced activation of both the Z and R promoters [18,19](Fig 7A). These results indicate that TAZ, and to a lesser extent YAP, activates the Z promoter in EBV-negative cells.

### YAP and TAZ activate the Z IE promoter via TEAD binding motifs

To further define the Zp promoter sequences required for YAP and TAZ activation *in vitro*, we compared the ability of co-transfected YAP and TAZ to activate a series of 5 ‘deletions of the Zp-346 promoter construct as depicted in Fig 7C. Examination of this region of Zp suggested three potential TEAD motifs between -218 and -266. As shown in Fig 7D, removal of two of the three TEAD motifs in the Zp-226 construct decreased YAP and TAZ activation by approximately 50%, and removal of all three TEAD motifs in the Zp-218 construct eliminated the ability of YAP and TAZ to activate Zp. In contrast, removal of the TEAD motifs did not inhibit the ability of co-transfected BLIMP1 to activate Zp (Fig 7E). These results suggest that both YAP and TAZ activate the Zp promoter though TEAD binding motifs. Since these TEAD binding motifs are located near to the Rp promoter (within about 2000 basepairs downstream of the transcription start site) in the intact EBV genome, they may also serve to mediate YAP/TAZ activation of Rp transcription in the context of the intact viral genome.

### YAP and TAZ are complexed to the EBV immediate-early gene region *in vivo*

**Fig 8:**
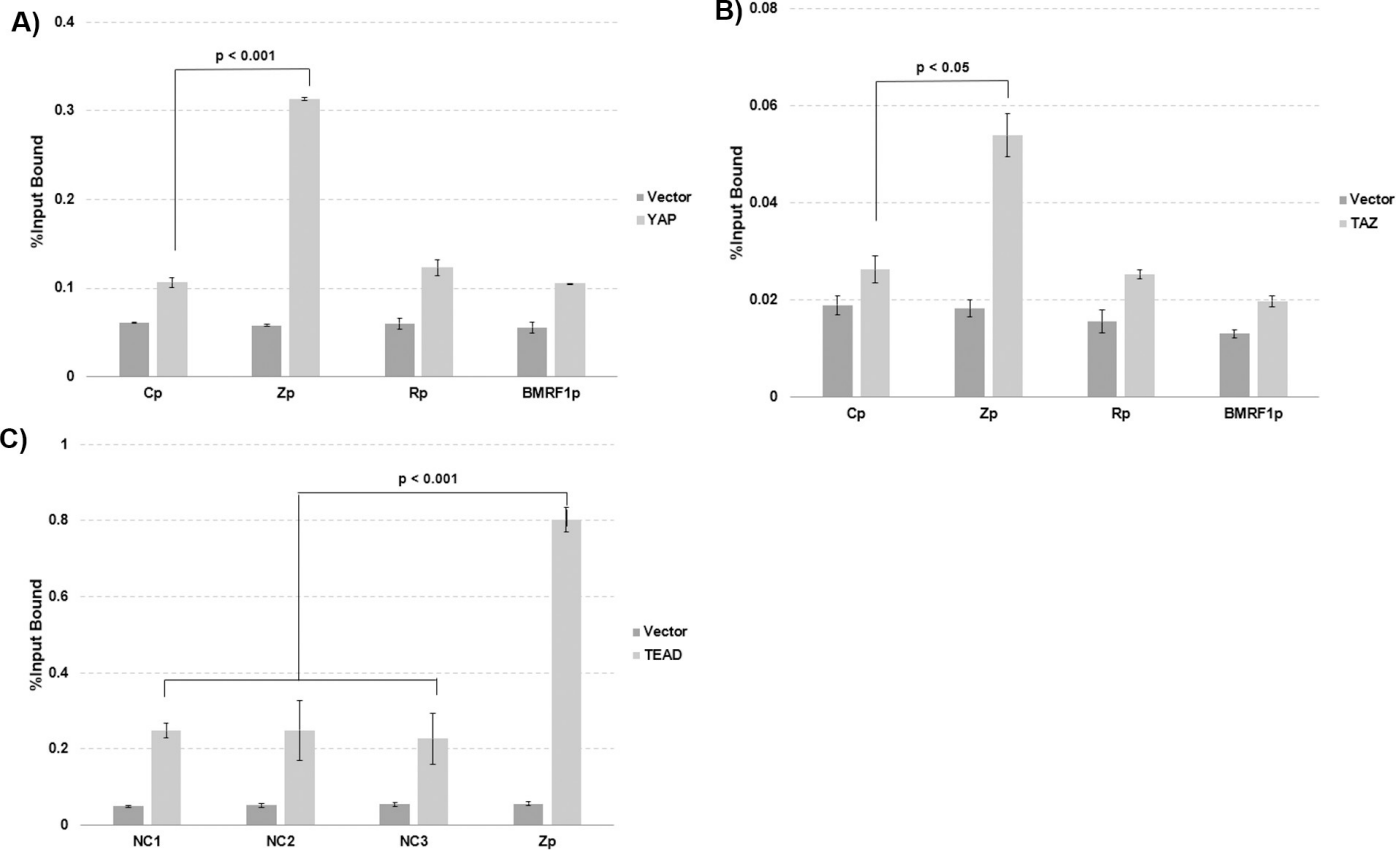
YAP and TAZ are complexed to the Z immediate-early promoter. HONE-Akata cells were transfected with either **A)** a vector control or co-transfected with a FLAG-tagged YAP(5SA) expression vector and TEAD1 vector, or **B)** a vector control or a FLAG-tagged TAZ(S89A) expression vector and TEAD1 vector. ChIP assays were performed 24 hours later using anti-FLAG antibody as described in the materials and methods. Binding of FLAG-tagged YAP(5SA) and TAZ(S89A) to various parts of the EBV genome, including the lytic Z, R and BMRF1 promoters and the latent Cp promoter was determined by qPCR. **C)** HONE-Akata cells were transfected with a vector control or FLAG-tagged YAP(5SA) expression vector and myc-tagged TEAD1 vector. ChIP assays were subsequently done 24 hours post transfection with a myc antibody as described in the materials and methods. Binding of myc-TEAD1 protein to the Z promoter, or negative control (NC) sites on the EBV genome was determined by qPCR. All experiments are representative of two independent biological replicates, and statistical significance was determined by the two-sample t-test.

To determine if YAP and/or TAZ can associate with the EBV genome IE region in EBV-infected cells *in vivo*, we performed ChIP assays in HONE-Akata cells co-transfected with TEAD1 and FLAG-tagged YAP(5SA) or FLAG-tagged TAZ(S89A) vectors, or control vectors, and then performed qPCR assays to assess YAP or TAZ association with various regions of the EBV genome. Both YAP and TAZ were found to be preferentially associated with the EBV Zp sequence compared to other regions of the EBV genome examined (including the R and BMRF1 lytic promoter sequences and the latent Cp promoter sequence) (Fig 8A and 8B). Transfected myc-tagged TEAD1 protein also preferentially associated with the EBV Zp sequence (compared to three other negative control regions of the EBV genome that lack local putative TEAD motifs) in ChIP assays (Fig 8C). These results suggest that YAP/TAZ are complexed to TEAD motifs in the YAP-responsive region of the EBV BZLF1 IE promoter *in vivo* and that direct TEAD binding to Zp is at least partially responsible for its ability to reactivate lytic EBV infection. Nevertheless, since the binding of the YAP/TAZ/TEAD proteins to Zp is somewhat weak, we cannot totally exclude the possibility that YAP/TAZ/TEAD also indirectly activate Zp by binding/activating a promoter driving an as yet unknown cellular transcription factor that activates Zp.

### YAP, TAZ, and TEADs are expressed at much higher levels in EBV-positive and EBV-negative epithelial cell lines in comparison to EBV-positive B cell lines

**Fig 9:**
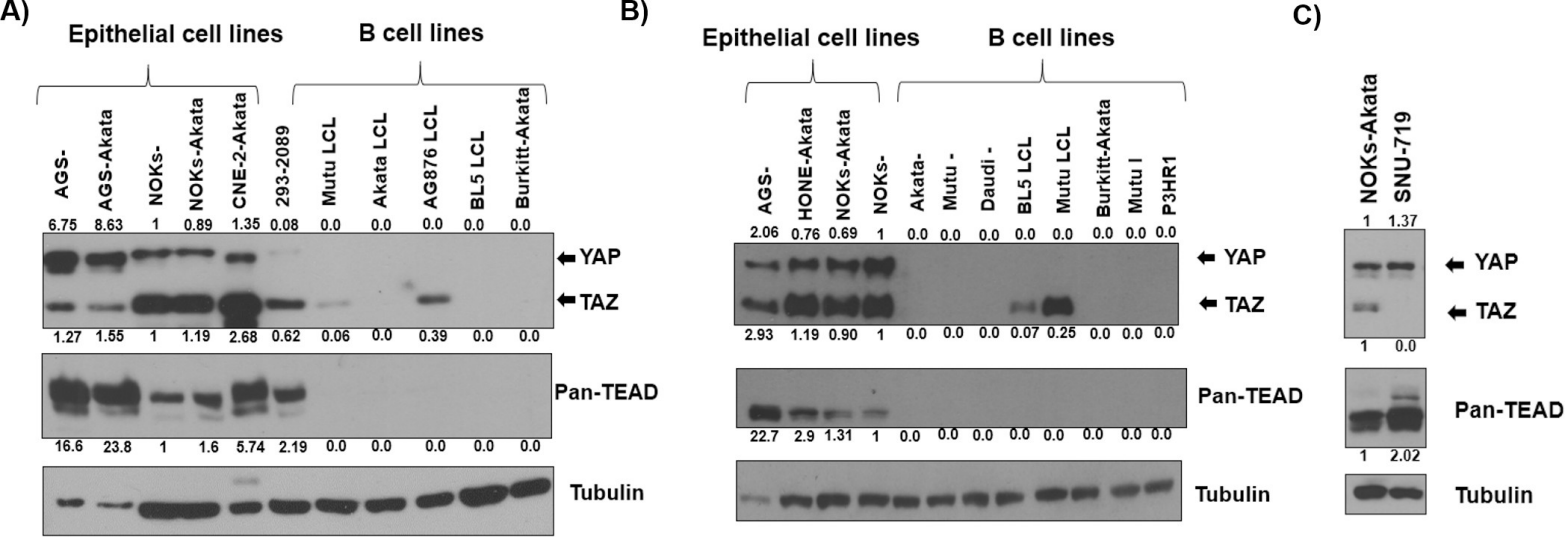
YAP, TAZ, and TEADs are expressed in EBV-infected gastric and oral epithelial cells but are not highly expressed in B cells. **A)** An immunoblot blot was performed to survey YAP/TAZ, TEADs, and tubulin expression in a number of different cell lines of either epithelial or B cell origin. Note that less protein was loaded in the AGS samples. The uninfected NOKs- cell condition result was set as 1. **B)** A repeat immunoblot experiment was performed in various epithelial cell lines and B cell lines in which expression of YAP/TAZ, TEADs, and tubulin was assessed. Note that less protein was loaded in the AGS sample. Results were quantitated using ImageJ software and normalized to the loading control result for each condition. The uninfected NOKs- cell condition result was set as 1. **C)** YAP/TAZ and TEAD levels were compared in NOKs-Akata versus SNU-719 gastric carcinoma cells. The results were quantitated using ImageJ software and normalized to tubulin for each condition. The NOKs-Akata cell condition was set as 1.

We next performed immunoblot analyses to assess the levels of total YAP and TAZ expression in various EBV-infected and uninfected epithelial cell lines. As shown in Fig 9A, 9B and 9C, although the ratio of YAP versus TAZ expression varied among the different cell lines, YAP and/or TAZ were found to be expressed in every epithelial cell line examined, including EBV-positive and EBV-negative AGS gastric carcinoma cells, EBV-infected SNU-719 gastric carcinoma cells, EBV-positive and EBV-negative NOKs cells, and EBV-positive CNE and HONE cells (cell lines originally thought to be NPCs but which are primarily composed of HeLa cells)[70]. EBV-infected 293 cells (a cell line currently thought to be derived from fetal mesenchymal stem cells) (48) also expressed some YAP and TAZ. EBV infection did not alter the total level of YAP or TAZ in either the AGS or NOKs cell lines.

We next examined the levels of total YAP and TAZ in four different EBV-infected B-cell lines, including two type 1 EBV-transformed lymphoblastoid cell lines (“Mutu” and “Akata”), two type 2 EBV-transformed lymphoblastoid cell lines (“AG876” and “BL5”) and one EBV-infected Burkitt lymphoma line (Akata BL) (Fig 9A and 9B). Interestingly, we found that none of the EBV-infected B cell lines express detectable YAP protein, and only a subset of the LCL lines express detectable TAZ. These results suggest that protein expression levels of YAP (and to some degree TAZ) are very cell type dependent and are very low in EBV-infected B cells.

We also compared the expression levels of TEADs in various different epithelial cell lines versus different B cell lines using a pan-TEADs antibody that detects all four TEAD family members. As shown in Fig 9A, 9B and 9C, we found that TEAD protein(s) are expressed in all epithelial cell lines surveyed (including EBV-infected NOKs, uninfected AGS cells, EBV-infected HONE and CNE cells, and EBV-infected SNU-719 cells). In contrast, we did not detect TEAD protein expression in any of the B cell lines, including three different EBV-negative Burkitt lymphoma lines (Daudi, Mutu and Akata), three different EBV-positive Burkitt lines (Akata, Mutu I and P3HR1) and four different EBV-transformed lymphoblastoid cell lines (Mutu, Akata, AG876 and BL5). These results indicate that specific signals that induce YAP/TAZ activity and lytic EBV reactivation in EBV-infected epithelial cells are unlikely to do so in EBV-infected B cells due to the generally extremely low levels of YAP, TEAD (and often TAZ) expression in this cell type.

### The combination of over-expressed TEADs, YAP and TAZ is sufficient to induce EBV lytic reactivation in an EBV-infected B cell line

**Fig 10:**
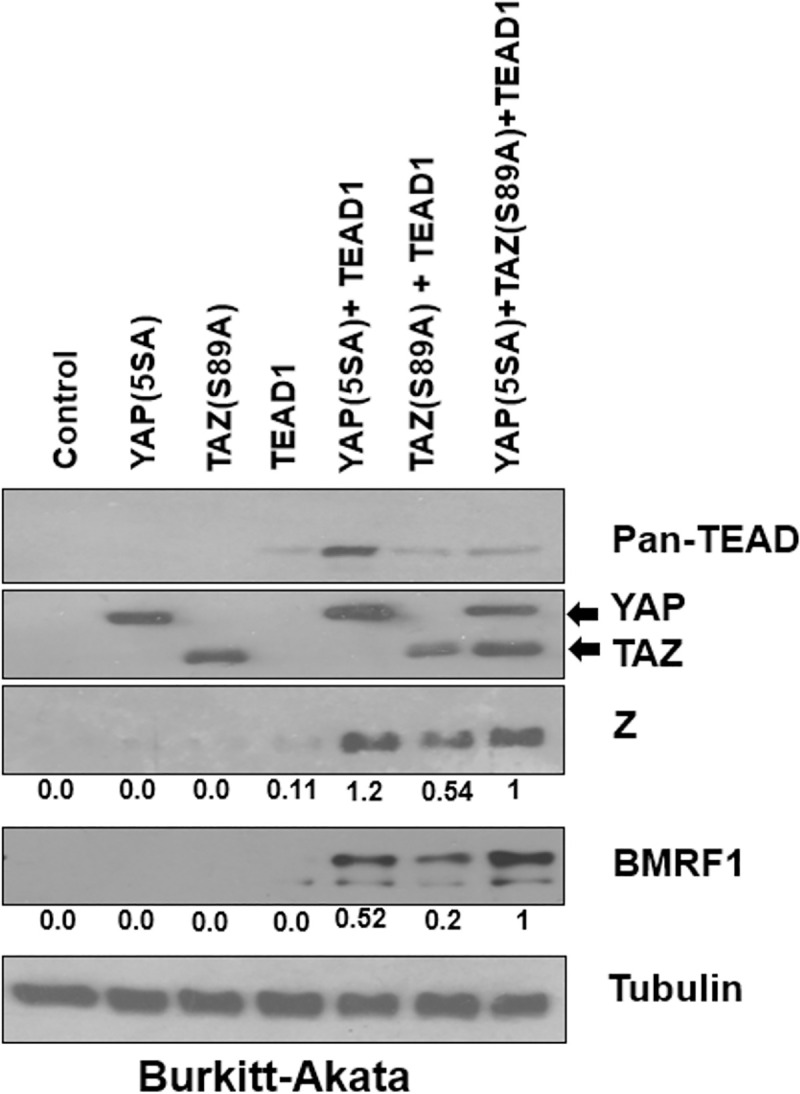
YAP and TAZ cooperate with TEADs to induce EBV lytic reactivation in B cell lines. Akata Burkitt-lymphoma cells were transfected with YAP(5SA), TAZ(S89A), and TEAD1, alone or in combination with each other, along with a vector control. After 48 hours post-transfection the cells were harvested and immunoblot performed to detect expression of YAP, TAZ, TEADs, Z, BMRF1 and the loading control tubulin. Results were quantitated using ImageJ software and normalized to the loading control result for each condition. The result in cells transfected with YAP(5SA), TAZ(S89A), and TEAD1 was set as 1.

We next investigated if YAP or TAZ over-expression, with or without co-transfected TEAD proteins, is sufficient to induce EBV lytic reactivation in a B-cell environment that does not normally express these transcriptional effectors. EBV+ Akata Burkitt lymphoma cells were transfected with YAP(5SA) and/or TAZ(S89A) expression vectors, with or without a TEAD1 expression vector and the levels of EBV lytic proteins, BZLF1 and BMRF1, was examined by immunoblot analysis 24 hours later. In the absence of co-transfected TEAD1, neither YAP(5SA) nor TAZ(S89A) expression vectors could induce lytic EBV protein expression (Figs 10 and S6). However, when either the YAP or TAZ vectors were co-transfected with the TEAD1 expression vector, lytic EBV protein expression was induced (Figs 10 and S6). These findings further confirm that YAP and TAZ cooperate with TEADs to induce lytic EBV reactivation and suggest that EBV-infected B cells are not intrinsically resistant to the lytic-inducing effects of these transcription factors, assuming they are expressed.

### EBV infection increases YAP/TAZ activity in NOKs cells

**Fig 11:**
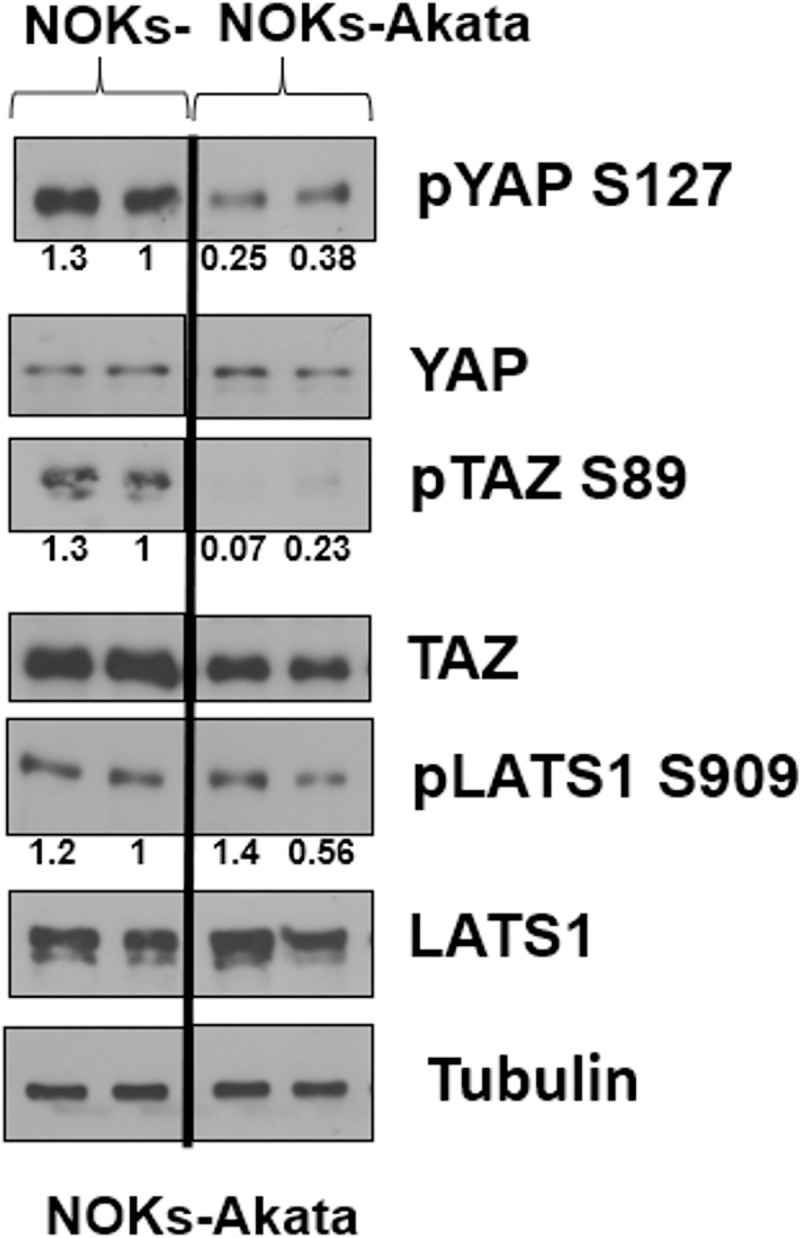
EBV infection of NOKs cells increases YAP and TAZ activity. EBV-negative and EBV-positive NOKs-Akata cells were grown in sub-confluent conditions in KSFM media without growth factors for 24 hours. The cells were then harvested for an immunoblot to examine expression of total YAP, TAZ, LATS1, YAP phosphorylated at serine reside 127, TAZ phosphorylated at serine residue 89, and LATS1 phosphorylated at serine 909. Tubulin expression was examined as a loading control. Results were quantitated using ImageJ software and normalized to the loading control result for each condition. The uninfected NOKs cell condition shown in lane 2 was set as 1. Black line indicates where irrelevant lane(s) in the western blot were removed.

To determine if the presence of EBV in epithelial cells affects the state of YAP or TAZ activation, we also compared the amount of activated (dephosphorylated) versus inactivated (phosphorylated) YAP and TAZ in EBV-infected versus uninfected NOKs cells. For these assays, cells were plated at sub-confluent density and grown in the absence of growth factors. Immunoblots were performed using antibodies that recognize total YAP or TAZ, versus antibodies that recognize inactivated YAP (phosphorylated at S127) or inactivated TAZ (phosphorylated at S89). We also examined the levels of phosphorylated (active) versus total LATS1 protein. As shown in Fig 11, the levels of YAP and TAZ phosphorylation were decreased in EBV-infected versus uninfected NOKs, suggesting that EBV infection increases YAP and TAZ activity in NOKs cells. Similar results were obtained in another experiment (S7 Fig). Interestingly, however, as shown in Fig 11, we did not find that EBV infection of NOKs cells affected the level of LATS1 phosphorylation (a modification which increases LATS1 function and thus leads to decreased YAP/TAZ phosphorylation), suggesting that EBV acts to inhibit YAP/TAZ phosphorylation through some other mechanism.

### Lysophosphatidic acid, a YAP/TAZ activator, induces lytic EBV reactivation through a YAP/TAZ-dependent mechanism

**Fig 12:**
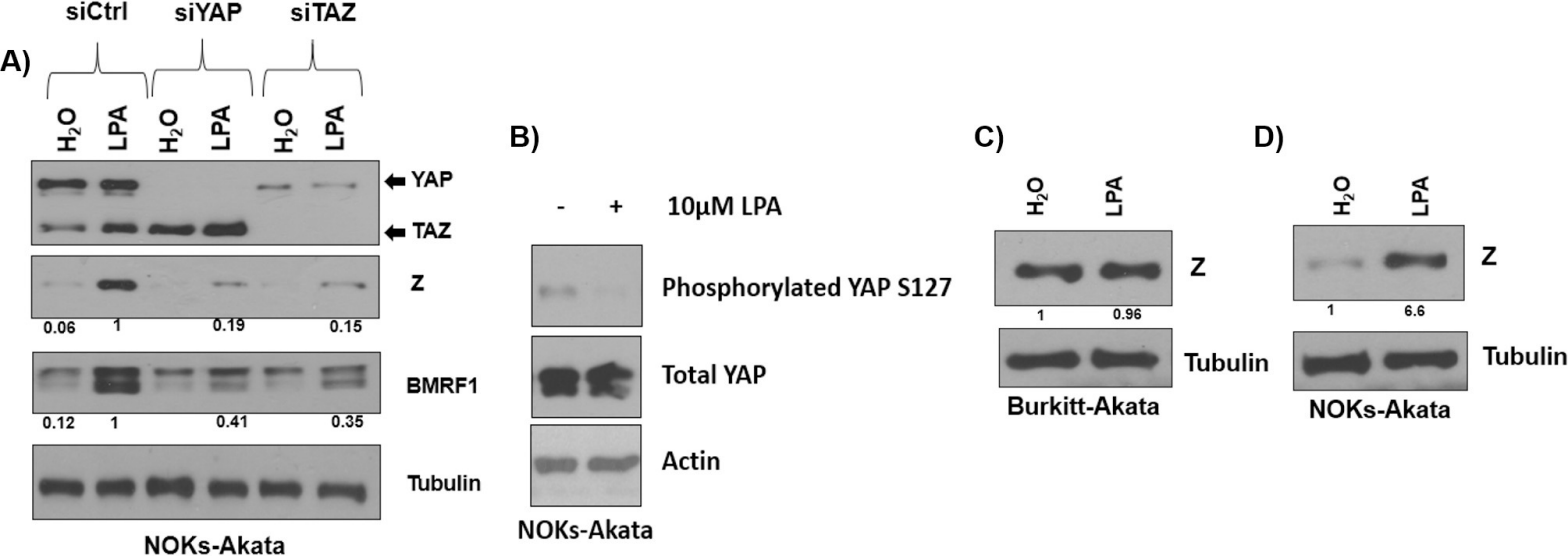
LPA induces EBV lytic reactivation through activating either YAP or TAZ in epithelial cells. **A)** NOKs-Akata cells were transfected with control siRNA or siRNAs targeting YAP or TAZ for 24 hours and then treated with 10 μM LPA for 24 hours before harvesting for a western blot. The expression of EBV lytic genes Z and BMRF1 was assessed, as well as the expression of YAP/TAZ, and tubulin for loading control. Results were quantitated using ImageJ software; and then normalized to the loading control result for each condition. The siRNA control result was set as 1. **B)** NOKs-Akata cells were treated with 10 μM LPA for 1 hour before harvesting for a western blot to assess the levels of total YAP versus YAP phosphorylated at serine residue 127. Actin level was assessed as a loading control. Results were quantitated using ImageJ software and then normalized to the loading control result for each condition. LPA treated NOKs-Akata cell results were set as 1. **C)** Burkitt-Akata cells were treated with 10 μM LPA for 24 hours before harvesting with lysis buffer to perform immunoblot analysis, where the expression of Z and the loading control tubulin was assessed. **D)** NOKs-Akata cells were treated with 10 μM LPA for 24 hours before harvesting for an immunoblot where the expression of Z and the loading control tubulin was assessed. Results were quantitated using ImageJ software and then normalized to the loading control result for each condition. The untreated cell results in each cell type were set as 1.

Lysophosphatidic acid (LPA), which is released by a variety of different cell types during inflammatory responses, binds to several different G-protein coupled receptors and induces YAP/TAZ activation by inhibiting LATS1/2 kinases [71,72]. To determine if LPA can induce lytic viral reactivation in EBV-infected NOKs in a YAP/TAZ-dependent manner, cells were treated for one day with control siRNA or siRNAs targeting YAP or TAZ and then treated with or without 10 μM LPA for another 24 hours. As shown in Figs 12A and S8, immunoblot analysis of the various conditions revealed that LPA treatment does indeed reactivate lytic EBV protein expression in NOKs-Akata cells, and that this effect is reduced when either YAP or TAZ expression is inhibited by siRNAs. We also confirmed that treatment of NOKs-Akata cells with LPA reduces the phosphorylation of YAP at serine 127, consistent with the previously described ability of LPA to activate YAP by inhibiting its phosphorylation (Fig 12B). In contrast, LPA does not induce lytic EBV reactivation in Burkitt Akata B cells (Fig 12C and 12D), which as shown in Fig 9 do not express TEAD, YAP or TAZ proteins. These results are the first to show that LPA induces EBV lytic reactivation in epithelial cells, and that this effect is mediated through activated YAP and TAZ. Thus, LPA is likely to be a biologically relevant stimulus by which YAP/TAZ activation can result in lytic EBV reactivation in humans.

## Discussion

EBV infection of B cells is generally latent, while infection of differentiated epithelial cells results in lytic infection. Stratified squamous oropharyngeal epithelial cells are a major site of lytic EBV infection in humans [7,9,73,74] and “rafted” oral keratinocytes provide a good *in vitro* system for modeling lytic “oral hairy leukoplakia” lesions that occur in differentiated tongue cells of immunosuppressed patients. We previously showed that differentiation-dependent expression of the cellular KLF4 and BLIMP1 transcription factors induces lytic EBV infection in stratified squamous oral epithelial cells by activating the Z and R IE EBV promoters [18,19]. EBV infection of pseudostratified respiratory nasopharyngeal epithelial cells (grown in air-liquid interface culture) also preferentially supports lytic EBV infection in differentiated suprabasal cells [39,40], although in this model system lytic EBV infection is not highly associated with either KLF4 or BLIMP1 expression [40] and some lytic gene expression occurs even in the undifferentiated basal cells [39]. Here we demonstrate that the Hippo effectors YAP and TAZ cooperate with TEADs to induce Z IE promoter activity and lytic EBV reactivation in epithelial cells, and show that the lack of YAP/TAZ/TEAD expression in B cells likely contributes to EBV latency in this cell type. Furthermore, we find that LPA, a phospholipid that activates YAP/TAZ function, promotes lytic EBV reactivation in epithelial cells via a YAP- and TAZ- dependent mechanism. Given the very high level of LPA in saliva, LPA may be a biologically important factor that serves to enhance lytic EBV infection in oropharyngeal epithelial cells independent of their differentiation state [75].

The differences in YAP, TAZ, and TEAD expression in epithelial cells versus B cells points to a previously unappreciated mechanism promoting lytic EBV infection in epithelial cells and latent viral infection in B cells [1,5,30]. In support of our results here, results presented in the Human Protein Atlas database indicate that RNA transcripts of YAP, TAZ, and the four TEAD family members (TEAD1, TEAD2, TEAD3, and TEAD4) are not detected in the EBV-infected Burkitt lymphoma cell line, Daudi [76]. Furthermore, the RNA transcripts of YAP, TEAD1, TEAD3, and TEAD4 are expressed at only extremely low levels in normal B cells in peripheral blood, although there is some low-level expression of TAZ and TEAD2 in normal B cells [76]. Further studies will be required to determine if EBV-encoded latency proteins further repress the already low levels of TAZ/TEAD2 protein expression in EBV-infected B cells, or whether certain stimuli can increase YAP/TAZ/TEAD expression in B cells and contribute to viral reactivation in this cell type.

The effects of activated YAP and TAZ are complex and very context- and cell type -dependent. While YAP and TAZ are known to induce cellular proliferation and support tumor formation in some contexts, there is emerging literature indicating that YAP and TAZ can also be essential for driving cellular differentiation in other contexts [46–48]. For example, the adenovirus E1A protein was recently shown to inhibit differentiation of 293 HEK cells into fibroblasts by sequestering YAP and TAZ in an inactive form in the cytoplasm [48]. In the case of oral epithelial cells, YAP and TAZ are reported to be expressed at highest levels in the undifferentiated basal cell layer and are thought to inhibit cellular differentiation [77–81]. Our finding here that YAP and TAZ are required for the ability of two different epithelial cell differentiation-inducing stimuli (collagen treated membrane/air-liquid interface culture and TPA treatment) to induce maximal lytic protein expression in EBV-infected epithelial cells (Figs 2 and 3) is thus somewhat paradoxical. However, since we did not observe any alterations in the ability of our NOKs cells to differentiate when YAP or TAZ was depleted with siRNAs (Fig 3), the ability of YAP and TAZ to induce lytic EBV protein expression appears to be distinct from their effects on epithelial cell differentiation. A similar paradox is the ability of ROCK inhibitor to prevent epithelial cell differentiation [81,82], even though ROCKs are required to induce YAP/TAZ activation in response to a number of different stimuli such as LPA, TGF-β, mechanotransduction and stress fiber formation [71,83–87]. Our results are consistent with a model in which low levels of active YAP/TAZ promote the initiation of lytic EBV reactivation in undifferentiated basal cell epithelium while not completely preventing epithelial cell differentiation.

YAP and TAZ do not bind to and activate target gene promoters independently and thus require DNA-binding co-factors for their transcriptional activity. Previous studies have reported that many co-activating partners, such as Runx2, ErbB4, p73, KLF4, and SMADs, can co-activate YAP and TAZ transcriptional targets, but the best-characterized partners are the four members of the TEAD family [53,54,58,59,88]. We determined that TEAD family members are essential for the ability of YAP and TAZ to induce lytic EBV reactivation, since the mutated YAP protein, YAP(5SA-S94A), which specifically cannot interact with TEADs, does not induce lytic reactivation, and co-transfection with a TEAD protein is required for the ability of both YAP and TAZ to induce lytic EBV reactivation in the Akata Burkitt lymphoma line (where YAP/TAZ and TEADs are not endogenously expressed) (Fig 10). Whether other potential mediators of YAP and TAZ transcriptional effects, in particular SMAD2/3 or KLF4, are also involved in their ability to induce lytic EBV reactivation remains to be determined in future studies.

EBV reactivation is initiated by cellular transcription factor-mediated activation of the two EBV immediate-early genes, BZLF1 and BRLF1. We find that the ability of TPA treatment, as well as growth of cells on collagen filters in air-liquid interface culture, to induce lytic EBV reactivation in epithelial cells requires expression of YAP and TAZ. We show that TAZ and YAP can activate the Zp (and to a lesser extent the Rp) in reporter gene assays and mapped a YAP- and TAZ-responsive motif in the Zp construct to several likely TEAD binding motifs located between -218 and -251 relative to the BZLF1 transcriptional initiation site. Since these motifs are within 2000 bp of the BRLF1 transcript start site in the context of the intact viral genome, we speculate that binding of TEADs with TAZ/YAP to this IE gene region in the context of the intact viral genome is sufficient to activate both the Zp and Rp. This is particularly likely to be the case given our previous finding that Z protein expression alone is not sufficient to induce lytic EBV reactivation in EBV-infected NOKs cells [24], and since we found that knock-down of YAP or TAZ in EBV-infected epithelial cells treated with either TPA or LPA similarly reduced Z and R expression.

Interestingly, we also found that the depletion of YAP and TAZ inhibits the ability of over-expressed transfected R IE protein to induce EBV lytic reactivation in NOKs-Akata cells (Fig 4A) and noted that this effect was accompanied by a large decrease in R-induced Z expression. Since both Z and R expression are required for induction of many early lytic genes, including the BMRF1 gene, in the context of the intact viral genome, our results suggest that YAP/TAZ may be primarily important for R activation of Zp. Consistent with this model, we found that the ability of the transfected Z and R proteins together to activate BMRF1 expression was not significantly inhibited by TAZ or YAP siRNAs (Fig 4B and 4C). R has been previously proposed to activate Zp indirectly through effects on cellular transcription factors including ATF-2 [89,90]. Our results here suggest that R activation of Zp may also be mediated by R modulation of YAP/TAZ activity, or at least require that YAP/TAZ are otherwise activated. Of note, we previously reported that R cannot induce lytic reactivation in EBV-infected B cell lines that do not already have some level of constitutive Z expression [23], which is intriguing given our findings here showing that B cells generally lack expression of TEAD family members and YAP/TAZ. Although we observed decreased YAP/TAZ phosphorylation in EBV-infected NOKs cells relative to the uninfected NOKs cells (suggesting enhanced YAP/YAZ function) (Fig 11), the EBV protein(s) mediating this activation remain to be determined in future studies.

Although YAP did not activate the Zp and Rp as efficiently as TAZ in reporter gene assays, we found that it is similar to TAZ in regard to its ability to reactivate EBV in latently infected cells. Interestingly, our siRNA experiments showed that both YAP and TAZ are individually required for the ability of TPA, R, LPA, and epithelial cell differentiation to induce lytic EBV reactivation in NOKs cells. Thus, the low level of TAZ (but not YAP) expression in EBV-infected SNU-719 gastric cancer cells (Fig 9C) may contribute to viral latency in this cell line. YAP and TAZ have around 40% amino acid conservation and have many homologous domains, and activate many of the same genes responsible for organ size, proliferation, and differentiation [46,48,50,57,91–93]. However, the cellular functions of YAP/TAZ are context- and tissue type-dependent [94]. For example, YAP expression is vital for the differentiation of gut epithelium, whereas TAZ is crucial for the differentiation of airway epithelium after injury [46,47]. YAP deletion in mice is embryonic lethal, while mice with a deletion of TAZ are viable [95,96]. Furthermore, the ability of 293 HEK cells to differentiate into fibroblasts following knock-down of adenovirus E1A protein was found to require both YAP and TAZ, and the YAP and TAZ regulated cellular genes were found to be only partially over-lapping in this differentiation process [48]. It will be interesting in future studies to determine why YAP and TAZ are both required for efficient lytic EBV reactivation in epithelial cells.

YAP, TAZ, and the TEADS are considered oncogenes and are overexpressed or amplified in numerous epithelial cancers [41–45]. TEADs are overexpressed 300-fold in Kaposi’s Sarcoma, and recent work has shown that lytic KSHV induces YAP expression [97,98]. The effect of EBV infection on YAP and TAZ function has not been well characterized in EBV infection, although one group has reported that LMP1 over-expression (outside the context of the viral genome) increases TAZ expression by interacting with the TAZ inhibitor gelsolin [99]. Additionally, TAZ has been reported to be expressed in the nuclear compartment of NPC tumor cells, suggesting that TAZ activation may play an oncogenic role during EBV-associated tumorigenesis [99]. In this study, we found that EBV-infected NOKs have decreased phosphorylation of YAP serine residue 127 and TAZ serine residue 89 compared to the uninfected NOKs. Since phosphorylation of YAP and TAZ at these sites prevents nuclear YAP/YAZ localization and inhibits cell growth [51], the ability of EBV to inhibit this phosphorylation in epithelial cells may play a role in the ability of EBV to inhibit epithelial cell differentiation [19,100,101] and/or promote epithelial cell tumors in humans. Although we did not observe a consistent effect of EBV infection in NOKs cells on the phosphorylation of the classic YAP and TAZ inhibitor, LATS1, numerous other mechanisms regulate YAP and TAZ phosphorylation, including various phosphatases such as PR55α that can directly remove these phosphorylation modifications [51,102]. Additionally, it is becoming increasingly clear that YAP/TAZ activity can also be activated through mechanisms not involving phosphorylation. For example, nuclear IRF3 and MRTF were recently shown to enhance YAP/TAZ transcriptional activity in a phosphorylation independent manner [60,103].

YAP and TAZ transcriptional activity is regulated by many upstream stimuli such as actin cytoskeleton remodeling, GPCR signaling, receptor tyrosine kinase signaling, cell-to-cell contact, WNT signaling, mechano-transduction, PKC activation, as well as AP-1 activity [67,71,84,85,88,104–106]. In this study, we investigated the ability LPA (which activates YAP/TAZ activity by inhibiting LATS1/2 phosphorylation) to induce lytic reactivation in EBV-infected cells. We chose to investigate the effect of LPA in particular since LPA is a biologically relevant stimulus present at very high levels in saliva [75,107]. We found that LPA does indeed induce lytic viral reactivation in EBV-infected NOKs cells at physiologically relevant levels, and that this effect requires both YAP and TAZ (Fig 12). Given that EBV is found at very high levels in periodontal lesions [108,109], and that LPA levels are particularly high in these lesions [75], we speculate that LPA may promote the replication of EBV in patients with gingival disease. It will clearly be important in future studies to investigate which of the other multitude of different YAP/TAZ activating pathways, in addition to LPA, can also contribute to lytic EBV reactivation, and in what contexts.

## Materials and methods

### Cell lines

The hTERT-immortalized oral epithelial NOKs cell line (a kind gift from Karl Munger, Tufts University) was derived as previously described [110] and was grown in KSFM supplemented with 0.1 μg epidermal growth factor (EGF) and 12.5 mg bovine pituitary extract (BPE) per 500 ml media. NOKs were infected with the Akata strain of EBV (containing a GFP marker and G418 resistance gene inserted into the viral BXLF1 gene) as previously described [24,111,112]. The AGS cell line is an EBV-negative gastric carcinoma cell line that was obtained from the ATCC and was grown in F-12 supplemented with 10% FBS and 1% pen-strep. The AGS-Akata cell line was infected with the Akata strain of EBV as previously described [111]. SNU-719 is an authentic EBV-infected gastric carcinoma cell line that was derived as described previously [68], and was maintained in RPMI media with 10% FBS and 1% pen-strep. HeLa is an HPV-positive cervical carcinoma cell line obtained from the ATCC and was maintained in DMEM media supplemented with 10% FBS and 1% pen-strep. EBV-positive and EBV-negative HONE and CNE cells (a gift from Lawrence Young, University of Birmingham) were originally described as EBV-negative nasopharyngeal carcinoma cell lines, but were subsequently shown to be contaminated with HPV-infected HeLa cells [70,113]. HONE and CNE cells were maintained in DMEM media supplemented with 10% FBS and 1% Pen-strep. HONE-Akata and CNE-Akata cells (each infected with the Akata strain of EBV) were grown in DMEM media supplemented with 10% FBS, 1% pen-strep and 400 μg/ml G418. The EBV-positive 293 cell line infected with EBV p2089 bacmid was described previously [114] and was grown in DMEM media supplemented with 10% FBS, 1% pen-strep, and maintained with 100 μg/ml hygromycin. The Akata, Mutu, BL5, and AG876 lymphoblastoid cell lines were derived by transforming peripheral B cells with each EBV strain as previously described [115], and were maintained in RPMI with 10% FBS and 1% Pen-strep. Akata-, Mutu-, and Daudi- are EBV-negative Burkitt lymphoma cell lines that were derived as previously described [116,117] (a kind gift from Kenzo Takada of Hokkaido University, Japan via Bill Sugden of the University of Wisconsin). Mutu-I is a Burkitt lymphoma cell line that was originally derived by the Alan Rickinson group at the University of Birmingham, UK, and was a kind gift from Jeff Sample of Penn State University [118]. P3HR-1 is a Burkitt lymphoma cell line [119], and was a kind gift from Bill Sugden of the University of Wisconsin. All Burkitt lymphoma cell lines were maintained in RPMI media with 10% FBS and 1% Pen-strep.

### Collagen membrane differentiation

Approximately 5x10^5^ NOKs-Akata cells were seeded onto a collagen-treated transwell membrane (Corning #3460) with KSFM media on both the basal and apical surfaces of the membrane. When cells seeded on the membrane were 100% confluent, all apical media was removed, and basal media was exchanged for Epilife media (Thermo Fisher #MEPICF500) supplemented with 10% FBS, 1.4mM CaCl_2_, and 5 μg/ml ascorbic acid. After three days, the membrane cells were harvested with sumo lysis buffer for immunoblot analysis.

### siRNAs

siRNAs against YAP (catalogue #sc-38637 and SASI_Hs01_00124477) and TAZ (catalogue #sc-38568B, #sc-38568C) were purchased from Santa Cruz and Millipore-Sigma, respectively. Millipore-Sigma’s Universal control (catalog# SIC001-1NMOL) and Santa Cruz siRNA controls A and C (catalog# sc-37007, sc-44231) were used as controls. siRNAs were used at 20 pM with 6 μL of RNAimax transfection reagent, and was delivered according to the RNAiMAX protocol (Invitrogen #13778150). After four hours post-transfection the media was changed to maintain cell viability, and the cells were harvested after two days post-transfection.

### DNA transfection

DNA was transfected into NOKs-Akata, NOKs, HONE-Akata, and AGS-Akata cells using the Lipofectamine 2000 (Thermo Fisher #11668019) system according to the manufacturer’s protocol. Generally, 500 ng of DNA total and 1.5 μl of Lipofectamine 2000 was used per condition to transfect epithelial cells that were approximately 70% confluent in a 12-well plate. Akata-Burkitt lymphoma cells were nucleofected using an Amaxa Nucleofector 2b device (Lonza) and program A-016 (with buffer V) with 1 μg of DNA in a 12-well plate. 48 hours after transfection, cells were washed with PBS and harvested with sumo lysis buffer for immunoblot analysis.

### Chemical reagents

Lysophosphatidic acid (LPA) was purchased from Thermo-Fisher, suspended in H_2_O at 10 mM and used at doses of 10 μM. Phorbol 12-myristate 13-acetate (TPA) was purchased from Sigma, and was suspended in DMSO for use at 20 ng/ml. Control conditions were treated equal amounts of the solvent.

### Plasmids

All plasmid DNA was prepared using the Qiagen Maxi-prep kit according to the manufacturer’s instructions. The plasmid pSG5 was purchased from Stratagene. The pSG5-R vector contains the EBV BRLF1 gene driven by the SV40 promoter (in pSG5) as previously described and was a gift from Diane Hayward at Johns Hopkins University [22]. The pCMV-FLAG YAP(5SA) plasmid (Addgene #27371), pCMV-FLAG YAP(5SA-S94A) plasmid (Addgene #33103), HA-TAZ(S89A) plasmid (Addgene #32840), pRK7-Myc-TEAD1 plasmid (Addgene #33109), and pRK7-Myc-TEAD2 plasmid were all gifts of Kun Liang-Guan at the University of California at San Diego. The 3XFLAG pCMV5-TOPO TAZ (S89A) plasmid (Addgene #24815) was a gift from Jeff Wrana at the University of Toronto. The HA-KLF4 plasmid (Addgene #34593 a gift from Michael Ruppert of West Virginia University) and BLIMP1 expression plasmid (a gift from Ken Wright of the University of South Florida) were described previously [18,19]. The pCpG Zp-83 luciferase, pCpG Zp-346 luciferase, pCpG Rp-1068, and pCpG BMRF1p luciferase expression vectors were all described previously [19].

### Construction of 5’ Z promoter deletions

5’ deletions were inserted into the pCpG Zp -346 luciferase construct using the following primers (with the name of the primer indicating the location in the Z promoter relative to the Zp transcriptional start site): Zp-266 primer ATGAAATCTTGGATACATTTCTAAATGA, Zp-226 primer GCATGCCATGCATATTTCAAC, Zp-218 primer TGCATATTTCAACTGGGCTGTCT. The primer TCGTCCAAATGCTGCAGG was the luciferase vector primer.

### Immunoblots

Immunoblots were conducted as previously described [120]. In brief, cellular extracts were harvested in sumo lysis buffer, and then proteins were run through a 10% polyacrylamide gel at 150 volts. Transfers to nitrocellulose were either done at 70 minutes at 100 volts or overnight at 22 volts. Once transfers were complete, a ponceau S stain was performed to assess transfer quality. 5% milk in wash buffer (1X PBS and .1% Tween-20) was used to block for an hour. Once blocking was completed, primary antibodies in either 5% milk or bovine serum albumin were added for either an hour or overnight depending on antibody requirements. After incubation, primary antibodies were removed and the membrane was washed 3 x 5 minutes before adding a secondary antibody for a period of one hour. The membrane was then washed again for 3 x 10 minutes before adding ECL (Thermo-Fisher) and imaging.

### Immunoblot analysis antibodies

Anti-YAP/TAZ (D24E4) dual antibodies were used at 1:1000 (Cell Signaling Technologies, catalog# 8418S), anti-YAP (D8H1X) antibody used at 1:1000 (Cell Signaling Technologies catalog# 14074T), anti-phosphorylated YAP S127 at 1:1000 (Cell Signaling Technologies, catalog# 4911), anti-TAZ (Novus, catalog# NB110-58359), anti-phosphorylated TAZ S89 (E1X9C) at 1:1000 (Cell Signaling Technologies, catalog# 59971), anti-BZLF1 antibody was used at 1:500 (Santa Cruz, catalog# sc-53904), anti-BRLF1 antibody at 1:2000 (isolated from rabbits injected with peptide sequence EDPDEETSSQAVKALREMAD), anti-BMRF1 antibody at 1:2000 (Millipore-Sigma, catalog# MAB8186), anti-LATS1 antibody at 1:1000 (Cell Signaling Technologies, catalog# 9153), anti-phosphorylated LATS S909 at 1:1000 (Cell Signaling Technologies catalog# 9157), PAN-TEAD antibodies at 1:1000 (Cell Signaling Technologies catalog# 13295S), anti-tubulin antibody at 1:4000 (Sigma, catalog# T5168), anti-β actin antibody at 1:5000 (Sigma catalog# 5441), and anti-HSP90 (F8) at 1:1000 (Santa Cruz catalog# sc-13119). The secondary antibodies used were Horseradish peroxide (HRP)- labeled goat anti-mouse antibody at 1:5000 (Thermo Scientific catalog# 31430), HRP- labeled donkey anti-goat antibody (Santa Cruz catalog# sc-2056, 1:5000), and HRP- labeled anti-rabbit antibody (Fisher Scientific catalog# 31460 1:10000).

### Luciferase reporter assays

Luciferase reporter assays were conducted as previously described[19]. 48 hours after transfection cells were washed once with PBS, suspended in 200 μl of 1X reporter lysis buffer (Promega) and then flash-frozen once. After pelleting by centrifugation and removal of the supernatant to a new tube, luciferase assays were performed according to the manufacturer’s instruction using a BD Monolight 3010 luminometer (BD Biosciences). All experiments were done in triplicate and repeated at least twice in separate experiments.

### ChIP assays

ChIP assays were performed as described previously [19]. For TEAD and YAP ChIP assays, HONE-Akata cells (using three 10cm dishes per condition) were transfected with a pcDNA empty vector control or pCMV-FLAG -YAP(5SA) plus myc-TEAD1 expression vectors. For the TAZ ChIP assays, HONE-Akata cells were transfected with the 3XFLAG-TOPO TAZ(S89A) and myc-TEAD1 expression vectors or pcDNA empty vector control. One day post-transfection, the cells were fixed with 1% paraformaldehyde for 10 minutes. After this period, the fixing reaction was quenched with 125 mM glycine for 5 minutes. The cells were then pelleted by centrifugation at 2000 rpm for 10 minutes, washed with PBS, and then spun again at 2000 rpm for 10 minutes. The cells were then harvested with cell lysis buffer (10mM Tris pH 8.0, 10mM NaCl, 0.2% NP40) with protease inhibitors (cOmplete, Roche) and left on ice for 10 minutes. The supernatant was then removed, nuclei lysis buffer (50mM Tris-HC pH 8.0, 10mM EDTA pH 8.0, 1% SDS) with protease inhibitors was added and the mixture was left on ice for 10 minutes before storing at -80°C overnight. The samples were then diluted in IP dilution buffer (20mM Tris-HCl pH 8.0, 2mM EDTA, 150mM NaCl, 1% Triton X100, and 0.01% SDS) and sonicated 4x30 seconds at 10 watts, with 90 seconds in between sonication (Fisher Scientific, Sonic Dismembrator Model 100). The samples were then blocked with magnetic A/G beads (Thermo-Fisher, 88802) for one hour. After blocking, immunoprecipitation was done with M2 FLAG (Sigma-Aldrich, M8823-1ml) beads overnight as directed by their protocol. For the myc-TEAD1 ChIP, immunoprecipitation was performed with myc-tag antibody (Cell Signaling Technologies catalog #2276 1:100) that was incubated overnight with the samples, and then mixed with the with magnetic A/G beads for two hours. The samples were then washed with low salt, high salt, and lithium chloride washes for 15 minutes each, followed by two 15-minute washes with T_10_E buffer. Samples then had elution buffer added to elute the DNA from the beads twice. Reverse cross-linking with 0.3M NaCl for 4 hours at 65°C. RNase A and proteinase K were added to all samples. Sample DNA was purified with phenol-chloroform, precipitated with EtOH overnight, and resuspended in T_10_E for real-time PCR.

### Real time PCR (qPCR)

Real-time quantitative PCR (qPCR) was conducted as previously described [19]. To briefly summarize, qPCR was conducted with an ABI Prism 7000 Sequence Detector with SYBR Green. All PCR reactions were done in a 96- well plate in either duplicate or triplicate by adding 2.5 μL sample, and 12.5 μL of SYBR Green PCR Master mix (Applied Biosystems, Foster City, CA, USA). The final concentration of primers was 0.3 μmol/L in a final volume of 25 μL. The PCR amplification protocol began at 50°C for 2 minutes followed by 10 minutes at 95°C and 40 PCR cycles consisting of 15 seconds at 95°C followed by 60°C for 1 minute. To ensure no genomic DNA contamination, each reaction contained an internal control, and separate water samples were run in tandem.

### qPCR primers

Primers used for qPCR were as follows: Z promoter ChIP forward: ATGGCATGCAGCAGACATTCATC, Z promoter ChIP reverse: AACACTAGAGTCCATGACAGAGGA, R promoter ChIP forward TGCCGGCTGACATGGATTACT, R promoter ChIP reverse GATGCTGATGCAGAGTCGCC. BMRF1 promoter ChIP forward: CACTGCGGTGGAGGTAGAG, BMRF1 promoter ChIP reverse: GGTGGTGTGCCATACAAGG, C promoter forward: GCCGTGGGAAAAAATTTATGG, C promoter reverse: CGCCAACAAGGTTCAATTTTCT, negative control (NC) 1—Forward (134221–134240: CACAGCTGCGTCTAGCCTTC)/ Reverse (134327–134346: AGTACAGCCGGTCGTAGTCA), NC2—Forward (62159–62178: TTCGCCGCGTTAAAAGCGTA)/ Reverse (62221–62239: GCTGGTGGCCGACACTTAT), NC3 -Forward (13387–13406: CAAGGGCGCCAGCTTTTCTC)/ Reverse (13440–13460: TGGGAGGCTGGACTTTACAGA); the negative control primers are named to reflect their locations in the Akata EBV genome KC207813.1.

## Supporting information

S1 Fig Depletion of YAP decreases constitutive lytic protein expression in AGS-Akata cells. Pooled siRNAs targeting YAP or a control sequence were transfected into AGS-Akata cells. Cells were harvested after two days and immunoblotted for Z, R, BMRF1, YAP, and tubulin (loading control). Results were quantitated using ImageJ software; results were normalized to the tubulin result for each condition. The siRNA control result was set as 1.
(TIF)

S2 Fig YAP and TAZ expression are both essential for efficient TPA-induced EBV lytic reactivation in NOKs cells. NOKs-Akata cells were treated with either 20 pM of two different control siRNAs, or 20 pM of two different siRNAs against TAZ or YAP. 24 hours post-siRNA treatment, the cells were dosed with 20 ng/ml TPA. After a subsequent 24 hours the cells were harvested for immunoblots, where the expression of YAP, TAZ, Z, R and BMRF1 was determined. Tubulin was used as a loading control. Results were quantitated using ImageJ software, with the results for each set of siRNAs averaged and then normalized to the tubulin result for each condition. The average siRNA control result was set as 1.
(TIF)

S3 Fig Efficient R-induced lytic reactivation requires expression of YAP and TAZ. **A)** NOKS-Akata cells were transfected with control siRNA or siRNA against YAP, and then 24 hours cells were transfected with an R expression vector or control vector. After another 24 hours an immunoblot was performed to assess the levels of R, YAP, Z, BMRF1, and actin. **B**) NOKS-Akata cells were transfected with control siRNA or siRNA against TAZ, and 24 hours later were transfected with R expression or control vectors. A western blot was performed to determine expression levels of TAZ, Z, R, BMRF1 and tubulin. **C**) NOKS-Akata cells were transfected with control siRNA or siRNAs against YAP, and were transfected 24 hours later with both Z and R expression vectors. The cells were harvested after 24 hours for immunoblot analysis where the expression of YAP, R, Z, BMRF1, and the loading control tubulin was determined. **D**) NOKS-Akata cells were transfected with control siRNA or siRNA against TAZ, and were then transfected 24 hours later with Z and R expression vectors. The cells were harvested after 24 hours for western blot analysis to detect expression of TAZ, R, Z, BMRF1, and HSP90. Results were quantitated using ImageJ software (with the results for each set of siRNA controls averaged) and normalized to the loading control result for each condition. The siRNA control result was set as 1. Black lines indicate where irrelevant lanes in the western blot were removed.
(TIF)

S4 Fig Constitutively activated YAP and TAZ induce lytic EBV reactivation in epithelial cell lines. **A)** AGS-Akata cells were transfected with constitutively active YAP (YAP(5SA)), or TAZ (TAZ(S89A)) expression vectors, or a vector control. Two days after transfection, the cells were harvested for a western blot and the expression levels of YAP, TAZ, Z, VCA-p18 and tubulin (loading control) was examined. Results were quantitated using ImageJ software and normalized to the loading control result for each condition. The vector control result was set as 1. **B)** NOKs-Akata cells were transfected with constitutively active YAP(5SA), TAZ(S89A), or a vector control. Three days after transfection the cells were harvested for an immunoblot where the expression of YAP, TAZ, Z, R, BMRF1, and tubulin was determined. Results were quantitated using ImageJ software and normalized to the loading control result for each condition. The second YAP(5SA) result was set as 1. **C)** SNU-719 cells were transfected with YAP(5SA) or TAZ(S89A) expression vectors, or a vector control, and immunoblot was performed to detect expression of transfected proteins YAP and TAZ, and Z, R, or tubulin (loading control). Results were quantitated using ImageJ software and normalized to the loading control result for each condition. The vector control result was set as 1.
(TIF)

S5 Fig YAP and TAZ cooperate with TEADs to induce EBV lytic reactivation in epithelial cells. **A)** NOKs-Akata cells were transfected with YAP(5SA-S94A), YAP(5SA), or control vectors. After 48 hours an immunoblot was performed to detect expression of Z, R, BMRF1, YAP, and tubulin. Results were quantitated using ImageJ software and normalized to the loading control result for each condition. The YAP(5SA) vector result was set as 1. **B)** HONE-Akata cells were transfected with the YAP(5SA) expression vector with or without a TEAD1 or TEAD2 expression vectors. 48 hours after transfection an immunoblot was performed to examine the expression of Z, R, BMRF1, TEADs and a tubulin loading control. Results were quantitated using ImageJ software and normalized to the loading control result for each condition. The YAP(5SA) vector plus TEAD1 result was set as 1.
(TIF)

S6 Fig YAP and TAZ cooperate with TEADs to induce EBV lytic reactivation in B cells. Akata Burkitt-lymphoma cells were transfected with YAP(5SA), TAZ(S89A), and TEAD1, alone or in combination with each other, along with a vector control. After 48 hours post-transfection the cells were harvested and immunoblot performed to detect expression of YAP, TAZ, TEADs, Z, BMRF1 and loading control tubulin. Results were quantitated using ImageJ software and normalized to the loading control result for each condition. The result in cells transfected with YAP(5SA), TAZ(S89A), and TEAD1 was set as 1.
(TIF)

S7 Fig EBV infection of NOKs cells increases YAP activity. EBV-negative and EBV-positive NOKs-Akata cells were grown in sub-confluent conditions in KSFM media without growth factors, in the presence or absence of LPA for 24 hours. The cells were then harvested for an immunoblot to examine expression of total YAP, YAP phosphorylated at serine reside 127 and tubulin. Results were quantitated using ImageJ software and normalized to the loading control result for each condition. The untreated uninfected NOKs cell condition shown in lane 1 was set as 1. Note that lanes 3 and 4 in this figure were also the source for phosphorylated and total YAP blot in Fig 12B.
(TIF)

S8 Fig LPA induces EBV lytic reactivation in epithelial cells in a YAP/TAZ dependent manner. NOKs-Akata cells were transfected with control siRNA or siRNAs targeting YAP or TAZ for 24 hours and then treated with 10 μM LPA for 24 hours before harvesting for a western blot. The expression of EBV lytic proteins Z and BMRF1 was assessed, as well as the expression of YAP/TAZ, and tubulin for a loading control. Results were quantitated using ImageJ software and then normalized to the loading control result for each condition. The siRNA control result was set as 1.
(TIF)
