# Supplementary figures and images for: Hippo signaling effectors YAP and TAZ induce Epstein-Barr Virus (EBV) lytic reactivation through TEADs in epithelial cells
Source: PLoS Pathog. 2021 Aug 2;17(8):e1009783. doi: 10.1371/journal.ppat.1009783 (PMC8360610; doi:10.1371/journal.ppat.1009783)

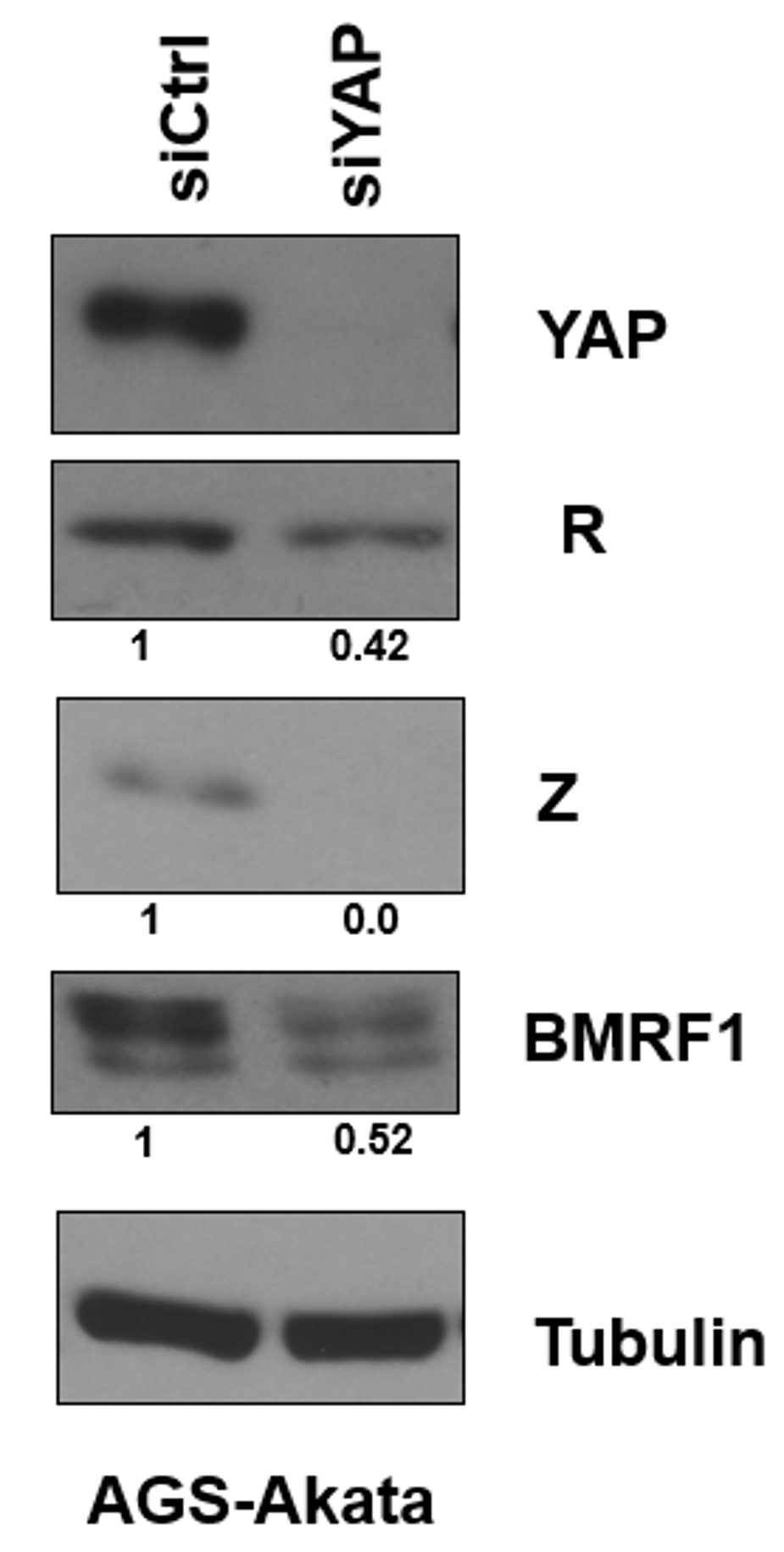

Supplement: S1 Fig — Pooled siRNAs targeting YAP or a control sequence were transfected into AGS-Akata cells. Cells were harvested after two days and immunoblotted for Z, R, BMRF1, YAP, and tubulin (loading control). Results were quantitated using ImageJ software; results were normalized to the tubulin result for each condition. The siRNA control result was set as 1. (TIF) [file ppat.1009783.s001.tif]

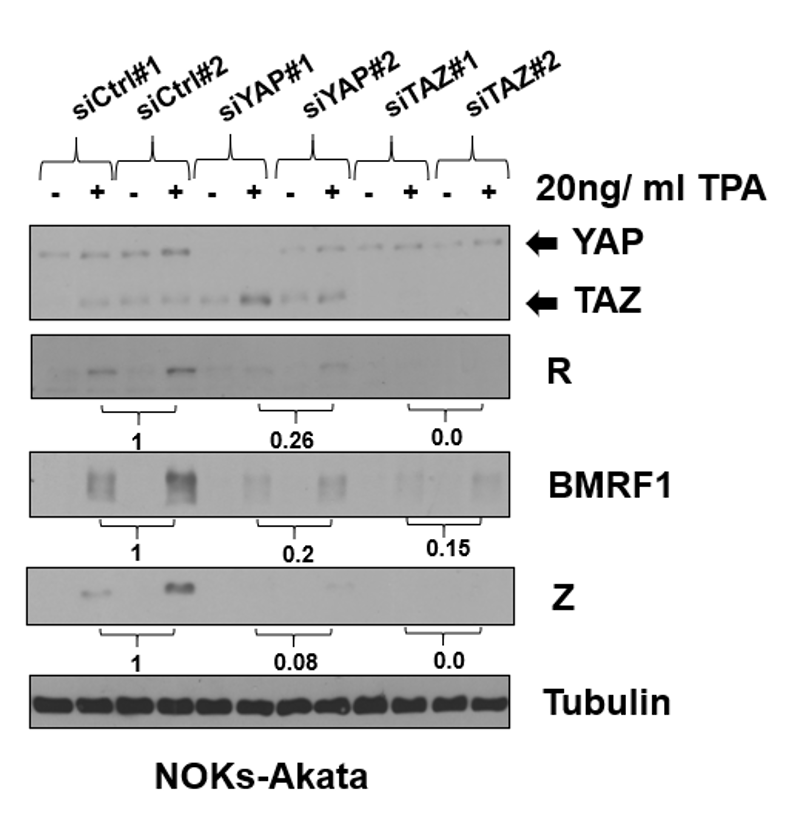

Supplement: S2 Fig — NOKs-Akata cells were treated with either 20 pM of two different control siRNAs, or 20 pM of two different siRNAs against TAZ or YAP. 24 hours post-siRNA treatment, the cells were dosed with 20 ng/ml TPA. After a subsequent 24 hours the cells were harvested for immunoblots, where the expression of YAP, TAZ, Z, R and BMRF1 was determined. Tubulin was used as a loading control. Results were quantitated using ImageJ software, with the results for each set of siRNAs averaged and then normalized to the tubulin result for each condition. The average siRNA control result was set as 1. (TIF) [file ppat.1009783.s002.tif]

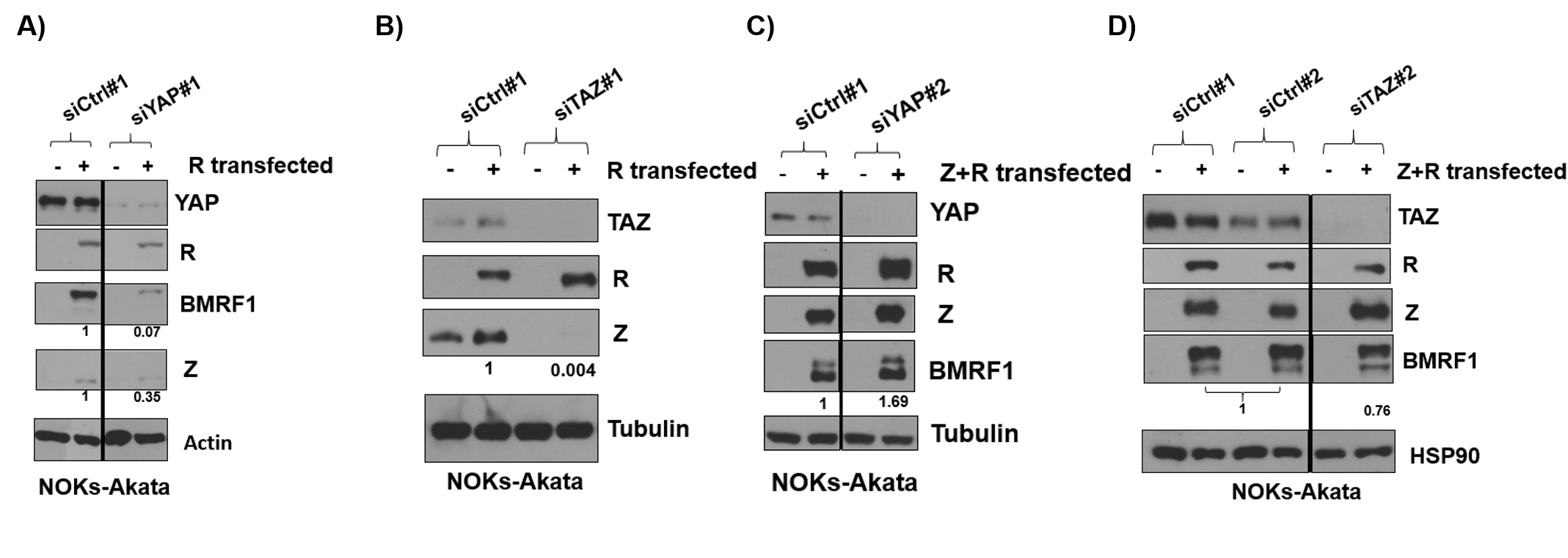

Supplement: S3 Fig — A) NOKS-Akata cells were transfected with control siRNA or siRNA against YAP, and then 24 hours cells were transfected with an R expression vector or control vector. After another 24 hours an immunoblot was performed to assess the levels of R, YAP, Z, BMRF1, and actin. B) NOKS-Akata cells were transfected with control siRNA or siRNA against TAZ, and 24 hours later were transfected with R expression or control vectors. A western blot was performed to determine expression levels of TAZ, Z, R, BMRF1 and tubulin. C) NOKS-Akata cells were transfected with control siRNA or siRNAs against YAP, and were transfected 24 hours later with both Z and R expression vectors. The cells were harvested after 24 hours for immunoblot analysis where the expression of YAP, R, Z, BMRF1, and the loading control tubulin was determined. D) NOKS-Akata cells were transfected with control siRNA or siRNA against TAZ, and were then transfected 24 hours later with Z and R expression vectors. The cells were harvested after 24 hours for western blot analysis to detect expression of TAZ, R, Z, BMRF1, and HSP90. Results were quantitated using ImageJ software (with the results for each set of siRNA controls averaged) and normalized to the loading control result for each condition. The siRNA control result was set as 1. Black lines indicate where irrelevant lanes in the western blot were removed. (TIF) [file ppat.1009783.s003.tif]

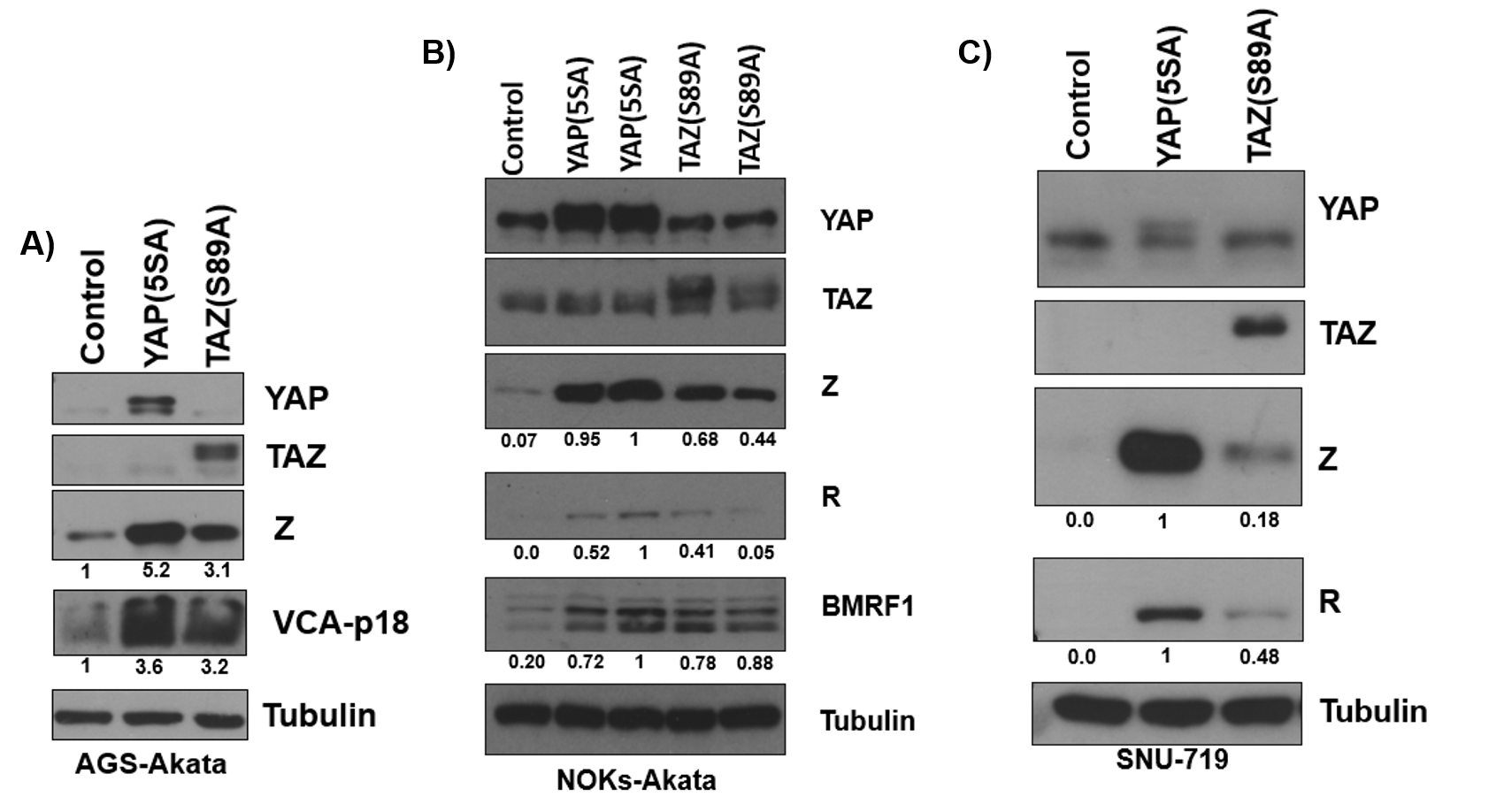

Supplement: S4 Fig — A) AGS-Akata cells were transfected with constitutively active YAP (YAP(5SA)), or TAZ (TAZ(S89A)) expression vectors, or a vector control. Two days after transfection, the cells were harvested for a western blot and the expression levels of YAP, TAZ, Z, VCA-p18 and tubulin (loading control) was examined. Results were quantitated using ImageJ software and normalized to the loading control result for each condition. The vector control result was set as 1. B) NOKs-Akata cells were transfected with constitutively active YAP(5SA), TAZ(S89A), or a vector control. Three days after transfection the cells were harvested for an immunoblot where the expression of YAP, TAZ, Z, R, BMRF1, and tubulin was determined. Results were quantitated using ImageJ software and normalized to the loading control result for each condition. The second YAP(5SA) result was set as 1. C) SNU-719 cells were transfected with YAP(5SA) or TAZ(S89A) expression vectors, or a vector control, and immunoblot was performed to detect expression of transfected proteins YAP and TAZ, and Z, R, or tubulin (loading control). Results were quantitated using ImageJ software and normalized to the loading control result for each condition. The vector control result was set as 1. (TIF) [file ppat.1009783.s004.tif]

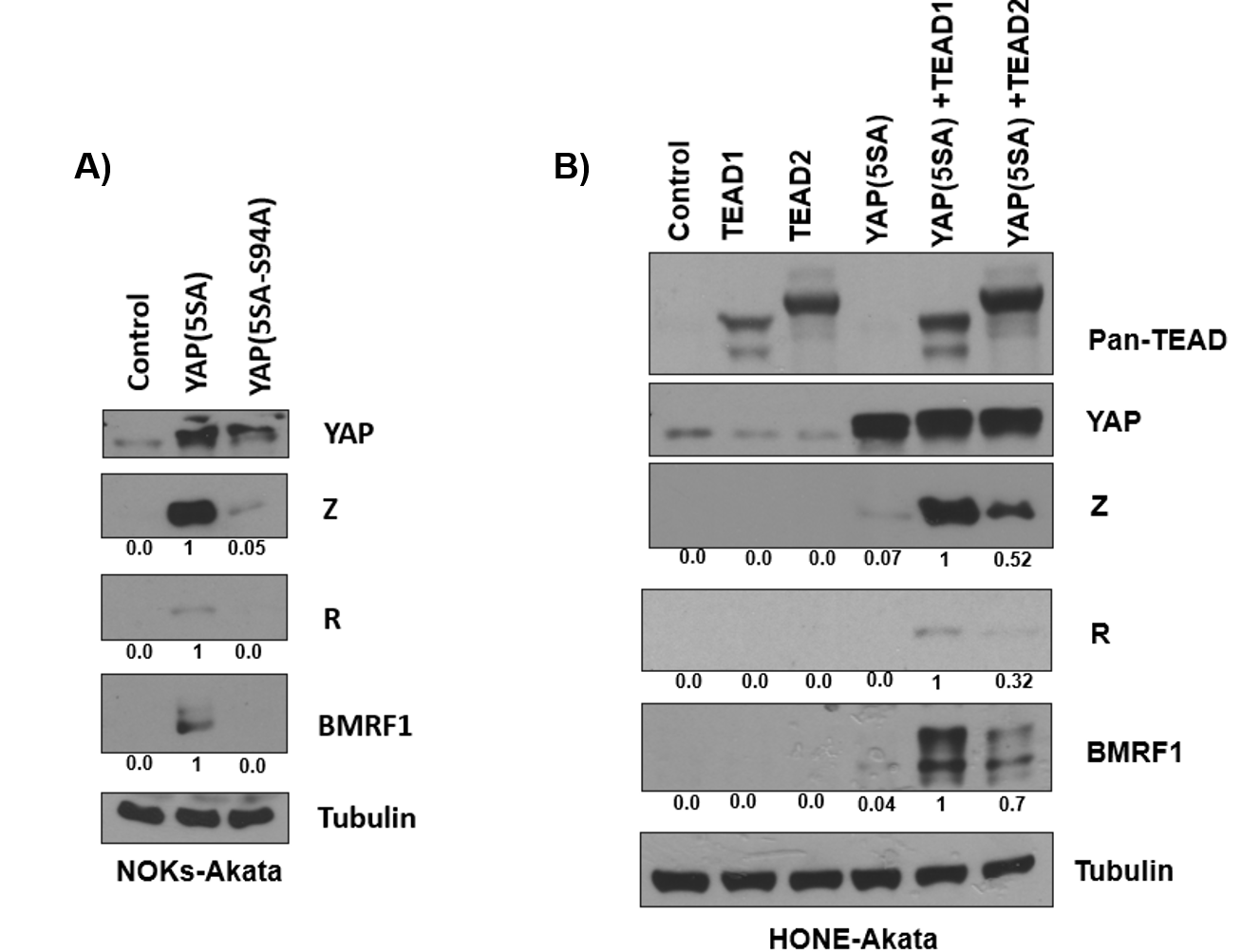

Supplement: S5 Fig — A) NOKs-Akata cells were transfected with YAP(5SA-S94A), YAP(5SA), or control vectors. After 48 hours an immunoblot was performed to detect expression of Z, R, BMRF1, YAP, and tubulin. Results were quantitated using ImageJ software and normalized to the loading control result for each condition. The YAP(5SA) vector result was set as 1. B) HONE-Akata cells were transfected with the YAP(5SA) expression vector with or without a TEAD1 or TEAD2 expression vectors. 48 hours after transfection an immunoblot was performed to examine the expression of Z, R, BMRF1, TEADs and a tubulin loading control. Results were quantitated using ImageJ software and normalized to the loading control result for each condition. The YAP(5SA) vector plus TEAD1 result was set as 1. (TIF) [file ppat.1009783.s005.tif]

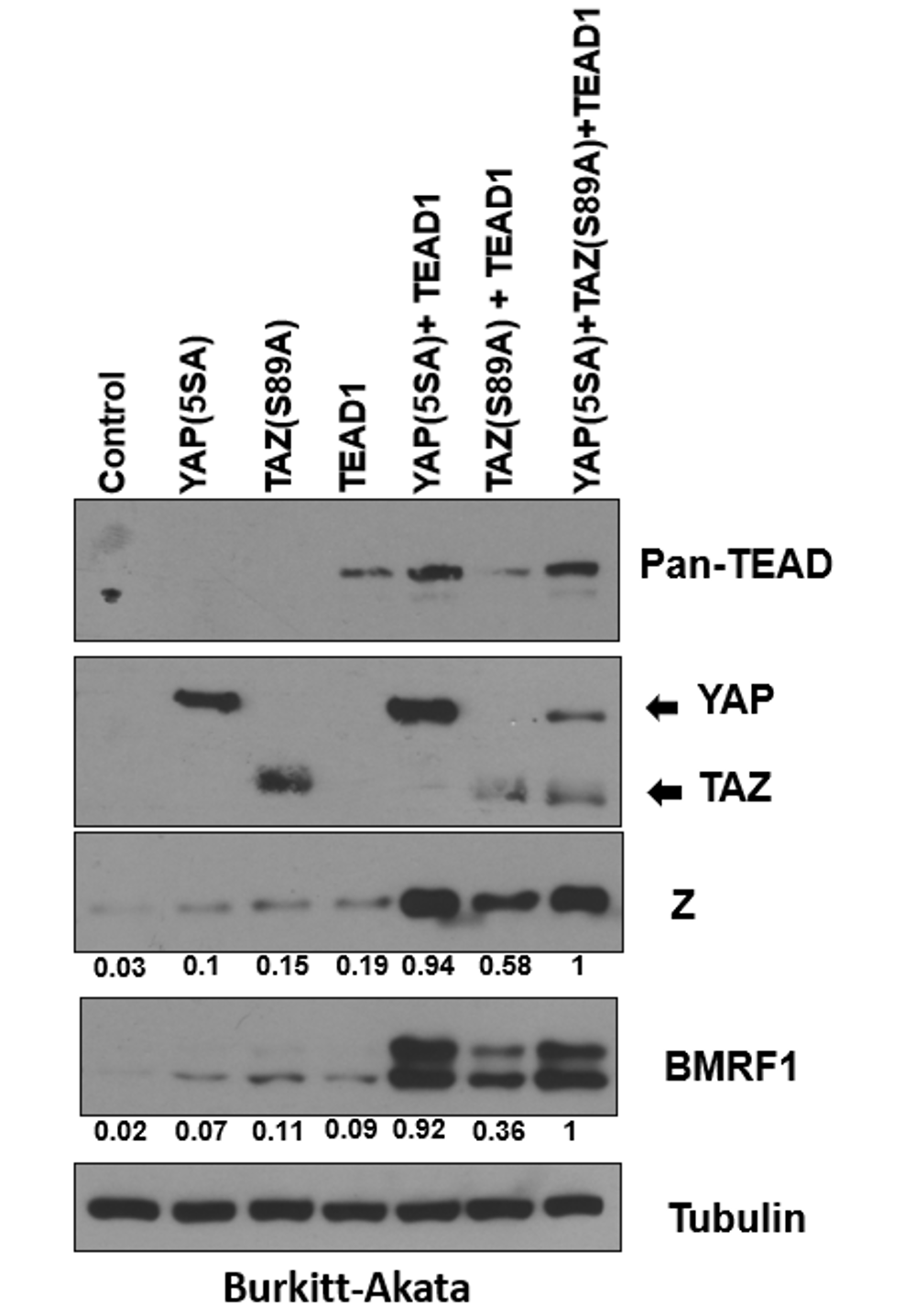

Supplement: S6 Fig — Akata Burkitt-lymphoma cells were transfected with YAP(5SA), TAZ(S89A), and TEAD1, alone or in combination with each other, along with a vector control. After 48 hours post-transfection the cells were harvested and immunoblot performed to detect expression of YAP, TAZ, TEADs, Z, BMRF1 and loading control tubulin. Results were quantitated using ImageJ software and normalized to the loading control result for each condition. The result in cells transfected with YAP(5SA), TAZ(S89A), and TEAD1 was set as 1. (TIF) [file ppat.1009783.s006.tif]

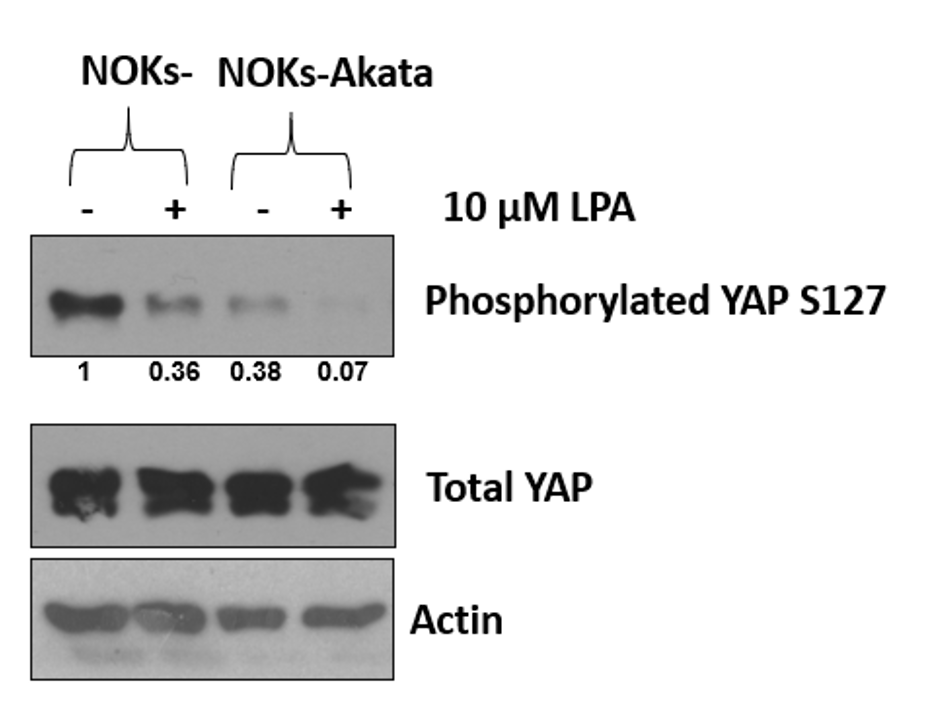

Supplement: S7 Fig — EBV-negative and EBV-positive NOKs-Akata cells were grown in sub-confluent conditions in KSFM media without growth factors, in the presence or absence of LPA for 24 hours. The cells were then harvested for an immunoblot to examine expression of total YAP, YAP phosphorylated at serine reside 127 and tubulin. Results were quantitated using ImageJ software and normalized to the loading control result for each condition. The untreated uninfected NOKs cell condition shown in lane 1 was set as 1. Note that lanes 3 and 4 in this figure were also the source for phosphorylated and total YAP blot in Fig 12B. (TIF) [file ppat.1009783.s007.tif]

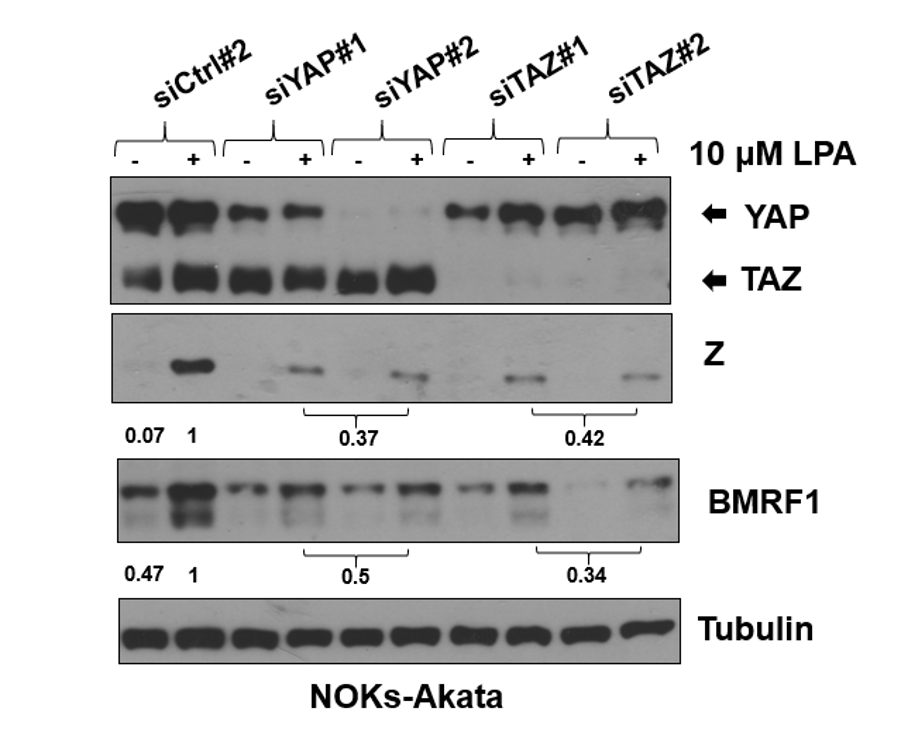

Supplement: S8 Fig — NOKs-Akata cells were transfected with control siRNA or siRNAs targeting YAP or TAZ for 24 hours and then treated with 10 μM LPA for 24 hours before harvesting for a western blot. The expression of EBV lytic proteins Z and BMRF1 was assessed, as well as the expression of YAP/TAZ, and tubulin for a loading control. Results were quantitated using ImageJ software and then normalized to the loading control result for each condition. The siRNA control result was set as 1. (TIF) [file ppat.1009783.s008.tif]
